# Novel Benzimidazole Derived Imine Ligand and Its Co(III) and Cu(II) Complexes as Anticancer Agents: Chemical Synthesis, DFT Studies, In Vitro and In Vivo Biological Investigations

**DOI:** 10.3390/ph16010125

**Published:** 2023-01-13

**Authors:** Prakasha G, H. D. Revanasiddappa, Jayalakshmi B, Prabhakar B. T, Chandan Shivamallu, Prashant M. Viswanath, Raghu Ram Achar, Ekaterina Silina, Victor Stupin, Natalia Manturova, Ali A. Shati, Mohammad Y. Alfaifi, Serag Eldin I. Elbehairi, Sanja J. Armaković, Stevan Armaković, Shiva Prasad Kollur

**Affiliations:** 1 Department of Studies in Chemistry, University of Mysore, Manasagangotri, Mysuru 570 006, Karnataka, India; 2 Department of Botany, Government College for Women, Mandya 571 401, Karnataka, India; 3 Molecular Biomedicine Laboratory, Postgraduate Department of Studies and Research in Biotechnology, Sahyadri Science College, Kuvempu University, Shimoga 577 203, Karnataka, India; 4 Department of Biotechnology and Bioinformatics, School of Life Sciences, JSS Academy of Higher Education and Research, Mysuru 570 015, Karnataka, India; 5 Centre for Excellence in Molecular Biology and Regenerative Medicine, Department of Biochemistry, JSS Medical College, JSS Academy of Higher Education & Research, Mysore 570 015, Karnataka, India; 6 Division of Biochemistry, School of Life Sciences, JSS Academy of Higher Education and Research, Mysuru 570 015, Karnataka, India; 7 Institute of Biodesign and Modeling of Complex Systems, I.M. Sechenov First Moscow State Medical University (Sechenov University), Moscow 119991, Russia; 8 Department of Hospital Surgery, N.I. Pirogov Russian National Research Medical University, Moscow 117997, Russia; 9 Biology Department, Faculty of Science, King Khalid University, Abha 9004, Saudi Arabia; 10 Cell Culture Lab, Egyptian Organization for Biological Products and Vaccines (VACSERA Holding Company), 51 Wezaret El-Zeraa St., Agouza, Giza 22311, Egypt; 11 Faculty of Sciences, Department of Chemistry, Biochemistry and Environmental Protection, University of Novi Sad, 21000 Novi Sad, Serbia; 12 Faculty of Sciences, Department of Physics, University of Novi Sad, 21000 Novi Sad, Serbia; 13 School of Physical Sciences, Amrita Vishwa Vidyapeetham, Mysuru Campus, Mysuru 570 026, Karnataka, India

**Keywords:** Schiff base, benzimidazole, DFT, antitumor activity

## Abstract

The emerging interest in the field of coordination chemistry and their biological applications has created a novel impact in the field of chemical biology. With this motivation, in this work we have synthesized a novel benzimidazole derived imine ligand, 2-((E)-((1H-benzo[d]-2-yl)methylimino)methyl)-4-fluorophenol (**HBMF**) and its Co(III) and Cu(II) complexes. The metal complexes (**C1**–**C4**) were synthesized in 2:1 (**HBMF**: metal ion) and 1:1:1 (**HBMF**: metal ion: 1,10-phen) ratios. Structural elucidations of all the synthesized compounds were performed using FT-IR, UV-Visible, NMR, Mass spectroscopy and elemental analysis techniques. A combination of first principles calculations and molecular dynamics simulations was applied to computationally investigate the structural, reactive, and spectroscopic properties of the newly synthesized **HBMF** ligand and its complexes with copper and cobalt metal ions. Quantum-mechanical calculations in this study were based on the density functional theory (DFT), while molecular dynamics (MD) simulations were based on the OPLS4 force field. The DFT calculations were used to obtain the reactive and spectroscopic properties of the ligand and its complexes, while molecular dynamics (MD) simulations were used to address the ligand’s reactivity with water. Further, the in vitro anti-proliferative activity of the compounds was tested against the A549, Ehrlich–Lettre ascites carcinoma (EAC), SIHA and NIH3T3 cell lines. The biological results depicted that the compound **C4**, with molecular formula C_27_H_23_Cl_2_CoFN_5_O_3_ exhibited profound anti-proliferative activity against the EAC cell line with a significant IC_50_ value of 10 µm when compared to its parent ligand and other remaining metal complexes under study. Various assays of hematological parameters (alkaline phosphate, creatinine, urea, RBC and WBC) were performed, and significant results were obtained from the experiments. Furthermore, the effect of **C4** on neovascularization was evaluated by stimulating the angiogenesis with rVEGF_165_, which was compared with non-tumor models. The EAC cells were cultured in vivo and administrated with 50 and 75 mg/kg of two doses and tumor parameters were evaluated.

## 1. Introduction

Schiff base is one of the privileged moieties in the field of medicinal biology with a wide array of pharmacological behavior [1]. Because of their synthetic flexibility, structural stability, and efficient coordinating entities with metal ions, they have been classified as a versatile ligand in the field of coordination chemistry [2]. Clinical trials of most chemotherapeutics display high toxicity due to poor ability in specificity towards cancerous cells [3]. The synchronization of antitumor activity found for several imine-based coordination complexes, includes target specification. Modification and modulation in the N-heterocycle containing Schiff base transition metal complexes is the one of the prime ways to study chemotherapeutic activities [4]. 

Over the last decade, much research has actively engaged in investigating copper and cobalt complexes, because of the assumption that they are endogenous metals which are less toxic. It has been widely established that the antitumor properties of copper and cobalt-coordinated compounds are largely determined by the nature of ligands and the donor atoms bound to the metal ion. The *N*-heterocycle containing imine base scaffolds with the modulated benzimidazole moiety served as a *N*-chelating skeleton for many transition metal ions [5]. Transition metal complexes fused with the benzimidazole imine base exhibit different pharmacologically diverse activity includes antibacterial, antifungal, anti-inflammatory, antioxidant and anticancer [6]. The platinum encapsulated metal complex in the field of chemotherapy has led to the scenario of the future transition metal complexes [7]. On the other hand, the chemistry of cobalt is fascinating in the field of co-ordination chemistry as its biochemistry is too vital and involves in the formation of RBC, synthesis of DNA, normalizing proper function of brain and nerve cells in the form of cobalamin and its derivatives [8]. Many research findings support the involvement of cobalt in amino acid and fatty acid metabolism [9]. This fact stimulates many researchers to remain hopeful for a cobalt incorporated imine base complex as an alternative to cisplatin with less toxicity [10]. 

Nowadays, modern atomistic calculations are unavoidable computational tools for identification of the best candidates for target applications in pharmaceutical and materials sciences, and for understanding of the underlying mechanisms of their activity [11,12]. In terms of the properties addressing the local reactivity, quantum-mechanical methods based on the density functional theory (DFT) have exhibited the most outstanding results when the accuracy-cost ratio is considered [13]. Besides quantum mechanical (QM) methods, molecular dynamics (MD) simulations have proven to be outstanding when it is necessary to explicitly involve solvent molecules [14]. This method also has shown advantages for studying extensive molecular systems consisting of many atoms. To obtain a clearer picture on the reactivity of newly synthetized molecular structures, it is always a good choice to perform calculations based on different methods and analyze different reactivity descriptors. Therefore, in this study, we performed a set of computational tasks to better understand the reactive properties of the newly synthetized ligand HBMF and its complexes with cobalt and copper.

The current research paper mainly focuses on the synthesis, spectral and pharmacological studies of 2-((E)-((1H-benzo[d]-2-yl)methylimino)methyl)-4-fluoropphenol (HBMF) and its Co(III) and Cu(II) complex. The detailed computational studies and investigation on the anticancer activity were carried out via in vivo and in vitro methods based on the IC_50_ values. The investigated complexes were also scanned for their DNA binding affinity and DNA incision property. 

## 2. Experimental

### 2.1. Materials and Methods

All the chemical reagents and solvents were directly used from a commercial source without any further purification. The 2-(aminomethyl)benzimidazoledihydrochloride, 5-fluorosalicydihyde and 1,10-phenanthroline monohydrates were procured from Sigma Aldrich Chemicals, Dresden, Germany. The melting point of the ligand and complexes were measured using the ELICO-3210 apparatus and are uncorrected. The elemental analyses (CHNS) of the compounds were determined on a Perkin–Elmer 240 elemental analyzer. The FT-IR spectra were recorded between 4000–400 cm^−1^ on a Bruker IFS66V spectrometer in KBr pallet (ligand) and Nujol-mull method complexes. The proton and carbon NMR was recorded on a Bruker NMR spectrometer using CDCl_3_ as a solvent and tetramethyl silane (TMS) as the internal standard. A Perkin–Elmer spectrometer was used to measure the Infrared spectra of the synthesized heterocyclic base and its complexes. An ELICO SL117 double beam spectrophotometer with a quartz cell was used to record the electronic spectra of novel ligand and its complexes. The copper complexes’ paramagnetic behavior (ESR) was scanned on a JEOL X-Band at 77 K under liquid nitrogen and TCNE (Tetracyanoethylene) as the g-marker. The mass spectra of the prepared compounds was recorded on a 2010 EV LC-MS Shimadzu spectrometer. The molar conductance measurement of the Schiff base metal complexes was performed with an Elico CM-180 conductometer having cell constant 1. The thermal analysis of the prepared complexes was done using a TGA-Q50 instrument heating rate of 10 °C /min and keeping the final temperature at 800 °C.

### 2.2. Preparation of 2-((E)-((1H-benzo[d]-2-yl)Methylimino)Methyl)-4-Fluoropphenol (HBMF)

**Scheme 1:**
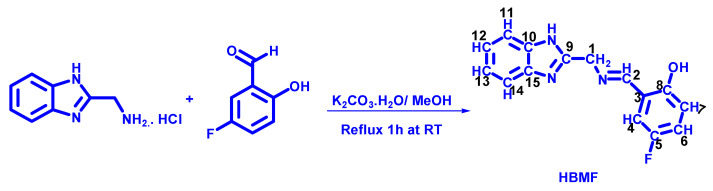
Synthetic pathway of Schiff base ligand (**HBMF**).

Scheme 1 depicts the general route for the preparation of HBMF. Neutralization of the aqueous solution of 2-(aminomethyl) benzimidazole hydrochloride (0.100 g, 0.4543 mmol) was done in the first step of synthesis using an aqueous solution of potassium carbonate (0.0753 g, 0.5451 mmol) and then mixed with a methanolic solution of 5-fluorosalicyladihyde (0.0630 g, 0.4543 mmol). The resulting mixture was stirred at room temperature using a magnetic stirrer. The yellow precipitate obtained was filtered off and washed with water followed by petroleum ether and dried under vacuum. The completion of the reaction was monitored on pre-coated silica gel plates by the thin layer chromatography (TLC) method using chloroform and methanol as the solvent system in the ratio (9:1). The Schiff base thus obtained was insoluble in water but soluble in methanol, DMSO, etc. Elemental analysis for **HBMF** (C_15_H_11_FN_3_O): Calculated (%): C, 62.91; H, 4.33; F, 7.05; N, 14.59; O, 5.90. Found (%): C, 62.95; H, 4.36; F, 7.09; N, 14.65; O, 5.94. ^1^H NMR (CDCl_3_, 400 MHz) ð: 5.09 [C1] (s, 2H, CH_2_); 6.86 [C7 and C6] (dd, 2H, Ar-H); 7.04–7.06 [C12 and C13] (t, 1H, Ar-H); 7.25 [C4] (d, 2H, Ar-H); 7.57 [C11] (d, 1H, Ar-H); 7.58 [C14] (d, 1H, Ar-H); 8.41 [C2] (s, 1H, N=CH); 12.80 (broad, 1H, Imidazole NH); (phenolic –OH not showing their peak due to high inductive effect of fluorine atom on aromatic system) (Figure S1). ^13^C NMR (CDCl_3_, 100 MHz) ð: 57.334 (C1), 115.95 (C11), 116.79 (C14), 117.02 (C7), 118.08 (C4), 118.15 (C12), 118.92 (C13), 120.17 (C6), 120.41 (C3), 122.96 (C10), 123.15 (C15), 150.27 (C9), 154.31 (C8), 156.78 (C5), 167.67 (C2) ppm (Figure S2). ESI-LCMS *m*/*z*: calcd. for C_15_H_11_FN_3_O), 270.27; found 271.110 (M + 1) (Figure S15).

### 2.3. Synthesis of Co(III) and Cu(II) Complexes in the Ratio 1:2 (C1 and C3)

The complexes (C1 and C3) were prepared by adding a hot methanolic solution of metal chlorides (CoCl_2_.6H_2_O and CuCl_2_⋅2H_2_O) to a methanolic solution of the ligand (**HBMF**) which was then refluxed for 6 h at 65 °C to get the required complexes of Co(III) and Cu(II) as a brown and green colored solid, respectively.

**C1**: [Cu(BMF)_2_]. Yield = 78%; m.p. 255–258 °C; green color; Anal (%) for C_30_H_22_CuF_2_N_6_O_2_: Calculated (%): C: 60.05, H: 3.66, N: 14.00, Cu: 10.59, F: 6.33 and O: 5.33. Found (%): C: 60.06, H: 3.69, N: 13.99, Cu: 10.58, F: 6.32 and O: 5.32.

**C3**: [Co(BMF)_2_]Cl Yield = 71%; m.p. 281–286 °C; brown color; Anal (%) for C_30_H_22_ClCoF_2_N_6_O_2_: Calculated (%): C: 57.14, H: 3.49, Cl: 5.55, Co: 9.36, F: 6.03, N: 13.33 and O: 5.07. Found (%): C: 57.13, H: 3.51, Cl: 5.61, Co: 9.33, F: 6.01, N: 13.31 and O: 5.06.

### 2.4. Synthesis of Co(III) and Cu(II) Complexes in the Ratio 1:1:1 (C2 and C4)

The complexes (C2 and C4) were synthesized by adding a hot methanolic solution of metal chlorides (CoCl_2_.6H_2_O and CuCl_2_⋅2H_2_O) to a methanolic solution of the ligand (**HBMF**) and 1,10-phenanthroline which was then refluxed at 65 °C for 6 h to get required complexes of Co(III) and Cu(II) as brown and green colored solid, respectively.

**C2**: [Cu(BMF)(phen)Cl]3H_2_O.Yield = 69%, m.p. 303–308 °C, green color, Anal (%) for C_30_H_25_ClCuFN_5_O_4_: Calculated (%): C: 55.01, H: 4.37, Cl: 6.02, Cu: 10.69, F: 3.16, N: 11.83 and O: 10.72. Found (%): C: 54. 04, H: 4.17, Cl: 5.88, Cu: 10.53, F: 3.14, N: 11.60 and O: 10.60.

**C4**: [Co(BMF)(phen)Cl]Cl·2H_2_O. Yield = 72% m.p. 311–316 °C, brown color, Anal (%) for C_27_H_23_Cl_2_CoFN_5_O_3_: Calculated (%): C: 52.97 H: 3.86, Cl: 11.59, Co: 9.77, F: 3.14, N: 11.51, O: 7.89. Found (%): C: 52.91, H: 3.76, Cl: 11.51, Co: 9.56, F: 3.08, N: 11.36, O: 7.78.

### 2.5. Pharmacology

The complexes were studied to assess their behavior at the cellular level and various interactions with cell constituent through in vitro and in vivo studies. The test compounds were primarily tested for their cytotoxicity and cell proliferation inhibiting action using the A459, EAC, SIHA and NIH3T3 cell lines (ATCC^®^ HTB-22™, Manassas, VA, USA). The results revealed that complex **C4** imparted potent anti-proliferative activity against the EAC cell lines, hence, further investigation of the complex **C4** for its anti-carcinogenic activity was carried out according to a previous report [15].

#### 2.5.1. Cell Culture and In Vitro Treatment

In vitro anti-proliferative assays of the cultured cell lines A549, EAC, SIHA and NIH3T3, under laboratory conditions, were performed. The cells were developed in Dulbecco’s Modified Eagle Medium (DMEM) (Gibco-Invitrogen, Austin, TX, USA), in addition with heat-inactivated Foetel Bovine Serum (Invitrogen, Waltham, MA, USA), Pen Strep and followed by the addition of NaHCO_3_ (0.40%). The resulting component was incubated at 37 °C and maintained at 97% humidity and 90% air atmosphere. Exactly 10 µM of the synthesized ligand **HBMF** and its complexes (**C1**–**C4**), prepared using DMSO as a solvent, was used initially to assess their cytotoxicity and anti-proliferative activity in vitro using the MTT assay as described previously [16]. 5-fluorouracil was used as a positive control and an appropriate vehicle control. 

#### 2.5.2. MTT Assay

The various cell lines, such as A549, EAC, SIHA and NI3T3, were used initially to investigate the extent of preliminary proliferation against the MTT assay. The synthesized ligand, the complexes, and 5-fluorouracil were treated with all four cell lines as mentioned above. The cells were treated in the absence and presence of the ligand and its complexes and were incubated for 2 days. The effect of the proliferating cells and the color change was investigated by adding 5 mg/mL of 3-(4,5-Dimethylthiazol-2-yl)-2,5-Diphenyltetrazolium Bromide (MTT) reagent [17].

#### 2.5.3. Animal Models and Ethics

Swiss albino male mice (weighed about 25 to 27 g) housed under standard laboratory conditions with food and water ad libitum. All the animal experimentation was performed after obtaining the permission from the Institutional Animal Ethics Committee, National College of pharmacy, Shimoga, India, in accordance with the Committee for the Purpose of Control and Supervision of Experiments on Animals (CPCSEA) guidelines for the laboratory animal facility (NCP/IAEC/101/05/2012-13).

#### 2.5.4. Hematological Studies

The standard methods viz., cell dilution fluids and hemocytometer were used to determine the hematological parameters such as WBC, RBC, alkaline phosphate, urea, and creatinine from normal mice as well as EAC cell bearing mice (ATCC^®^ HTB-18™, MYS, KA, IN). For this purpose, mice were grouped into 10 (*n* = 4), of five groups and tumors were transplanted and treatments were started immediately after 24 h. Group 1 mice were kept as untreated, Group 2 received a standard drug Bleomycin intraperitoneal (i.p.) (0.3 mg/kg, i.p.) and Groups 3–5 were treated with complex **C4** at an incremental doses, 2.5 mg/kg (i.p.), 5.0 mg/kg (i.p.) and 10 mg/kg (i.p.), respectively for each day per mouse continued for 10 consecutive days. At the interval of 5 days, the blood parameters were assayed [18]. The rest of the five groups of mice without tumors were used for studying the effect of the complex **C4** on hematological parameters. Group 1 mice were assayed without any treatment and groups 2–4 were treated with the complex **C4** at an incremental dose, 2.5 mg/kg (i.p.), 5.0 mg/kg (i.p.) and 10 mg/kg (i.p.), respectively, per day per mouse. A standard drug Bleomycin (0.3 mg/kg, i.p.) was injected to group 5.

#### 2.5.5. Animal Tumor Models and Treatment

The antitumor activity of the mixed ligand cobalt complex (**C4**) was tested against the EAC cells (Accession number: PF0041) in vivo (*n* = 30). The peritoneal cavity of the donor mouse with allowed multiply were injected with a definite number of Ehrlich Ascites carcinoma cells (1 × 10^−6^ cells per animal) for developing a liquid tumor of EAC. About 40 mice were included for study and divided into group A, group B, group C, and group D. A 1% solvent of DMSO was used to dissolve the complex **C4** and intraperitoneally (i.p) was injected by three doses on alternative days to group C mice at 50 mg/kg concentration and group D mice at 75 mg/kg concentration using 26-gauge needle as a body weight. Group B served as an appropriate vehicle control (DMSO). The survivability of the animals (*n* = 40) was monitored periodically.

#### 2.5.6. Chorioallontoic Membrane (CAM) Assay

The in vivo anti-angiogenic efficacy induced by rVEGF_165_ was analyzed on treatment with the complex **C4** (50 mg/kg and 75 mg/kg) in 15 days fertilized egg CAM in accordance with the method described previously [19] and the MVD changes were analyzed and photographed using a Sony steady shot DSC-W610 camera. 

#### 2.5.7. Measure of Micro-Vessel Density (MVD)

The density of the micro-vessel was analyzed by the in vivo peritoneal angiogenesis and H&E staining as described earlier [19]. In outline, the angiogenesis suppression in the peritoneum of tumor bearing mice subjected in the absence or presence of complex **C4** was photographed. With the help of light microscopy, the peritoneum of formaldehyde-fixed tissue was performed for H&E staining for the investigation of MVD [20].

### 2.6. Computational Details

The HBMF molecule and its complexes with Cu and Co were computationally studied employing DFT, TD-DFT calculations and MD simulations. First, the structure of the HBMF was subjected to conformational analysis employing the OPLS4 force field, using the workflow implemented in the MacroModel program [21]. All generated conformers were subjected to optimizations using the B3LYP density functional [22] and 6–31 G(d,p) basis set [23]. The 10 lowest energy conformations were re-optimized at the same level of theory, but with improved self-consistent field accuracy. Once the lowest energy conformer was identified, it was subjected to further calculations.

The initial structures of the complexes of HBMF were created with the 2D drawing tool of the Maestro program. Generation of the 3D structure was followed by force field minimization of fragments to obtain reasonable bond lengths and guess structures for geometrical optimizations at the QM level. Initial guess structures were subjected to geometrical optimizations using the extended tight binding (xTB) model and xTB program developed by Prof. Grimme and coworkers [24]. Input files for xTB calculations were made using the atomistica.online xTB input generator, while the xTB calculations were performed online using the corresponding tool available at https://atomistica.online, accessed on 20 November 2022 [25]. The structures of complexes were then re-optimized using the B3LYP density functional and MIDIXL basis set [26]. Frequency calculations at corresponding levels of theory were performed after geometrical optimizations to check that the geometrical optimizations had yielded the true ground states. Only positive values of frequencies were obtained, indicating that the true ground states were obtained.

In this work, all DFT calculations on organometallics were performed using the ligand field theory with d-d repulsion to construct the initial guess for the metal d orbitals. Only one possible state for the occupation of the d orbitals below the threshold of 0.01 Hartree was identified in all cases. In all cases of organometallics, doublet multiplicity was considered. In each complex, formal charges of metals, first and second ligands were +2, −1, and −1, respectively.

Optimized geometries of the ligand and its complexes were further subjected to DFT calculations using the M06-2X density functional [27] and LACVP(d,p) basis set [28], with the aim to obtain information on frontier molecular orbitals and quantum molecular descriptors such as molecular electrostatic potential (MEP) and average local ionization energy (ALIE). Time-dependent DFT (TD-DFT) calculations were performed with CAM-B3LYP [29] density functional. The MD simulations were performed with an OPLS4 force field [30] using the simulation time of 10 ns in an NPT particle ensemble under normal conditions. One ligand molecule was placed into the cubic simulation box surrounded by approximately 2000 water molecules described via the charge (SPC) model [31]. The obtained trajectories were used later to calculate radial distribution functions (RDF).

The DFT and TD-DFT calculations were performed with the Jaguar program [32,33], while the MD simulations were performed with the Desmond program [34]. Visualization was performed with the Maestro program [34]. Maestro, MacroModel, Jaguar and Desmond programs were used as implemented in the Schrödinger Materials Science Suite 2022-2.

## 3. Results and Discussion

### 3.1. Chemistry

**Figure 1:**
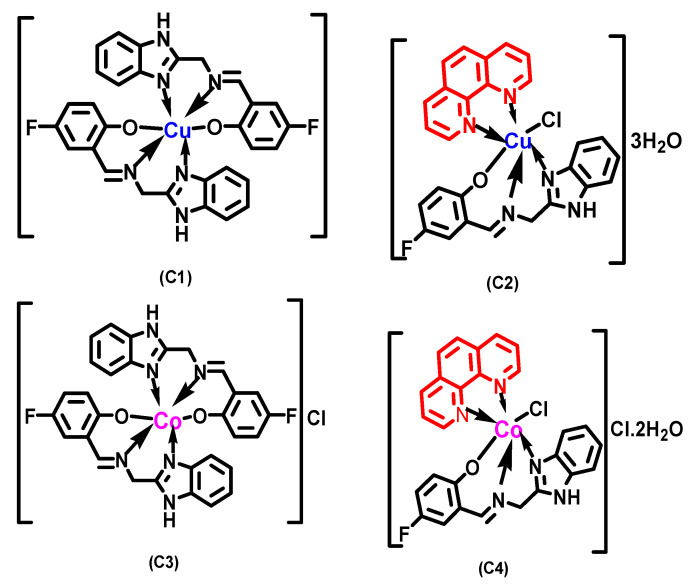
Proposed structures of metal complexes (**C1**–**C4**).

All the prepared complexes were stable at room temperature, soluble in 1% of DMSO, but the ligand was soluble in methanol and the complexes were soluble in DMF solvents. Looking at the results of the elemental analyses, the prepared metal complexes were in the 1:2 ratio (M: 2HBMF) and 1:1:1 ratio (M: **HBMF**: 1,10-phen) and their proposed molecular structures are depicted in Figure 1.

#### 3.1.1. Electronic Spectral Analysis

The UV-Visible spectra of all the synthesized compounds are showed in (Figures S3–S8). In the range of 305–330 nm, a weak absorption band was observed due to the azomethine linkage. The complexes **C3** and **C4** showed a band at 650 nm due to ^1^A_1g_→^1^T_2g_ transition [35,36]. All the complexes showed a common band at 390–435 nm suggesting that there was a possibility of charge transfer transition. The electronic spectra of complexes **C1** and **C2** clearly indicated a distorted octahedral geometry due to a band registered at 455 nm due to the ^2^B_1g_→^2^E_g_ transition. The complexes **C2** and **C4** contains the co-ligand, 1,10-phenanthroline, which affects the electronic absorption and that includes one more band at 360 nm due to the n→π* transition [37]. 

#### 3.1.2. IR Spectral Studies

In the FT-IR spectrum of **HMBF**, the benzimidazole ring shows a medium intense band at 1730 cm^−1^ due to the presence of a heterocyclic C=N (Figures S9–S14). A band observed at 1638 cm^−1^ indicates the free azomethine group in the Schiff base ligand (**HBMF**) but, in the case of the resultant complexes, there is a shift in this band towards a lower value, which strongly suggests that azomethine nitrogen is involved in the coordination [38]. The stretching frequency observed at 1270 cm^−1^ in **HBMF** was attributed to the phenolic (C-O) moiety, which was shifted to a lower wave number by 8–12 cm^−1^ in the spectra of the metal complexes. This observation unambiguously confirms the involvement of phenolic OH in the coordination [39].

#### 3.1.3. Thermogravimetric Analysis

**Figure 2:**
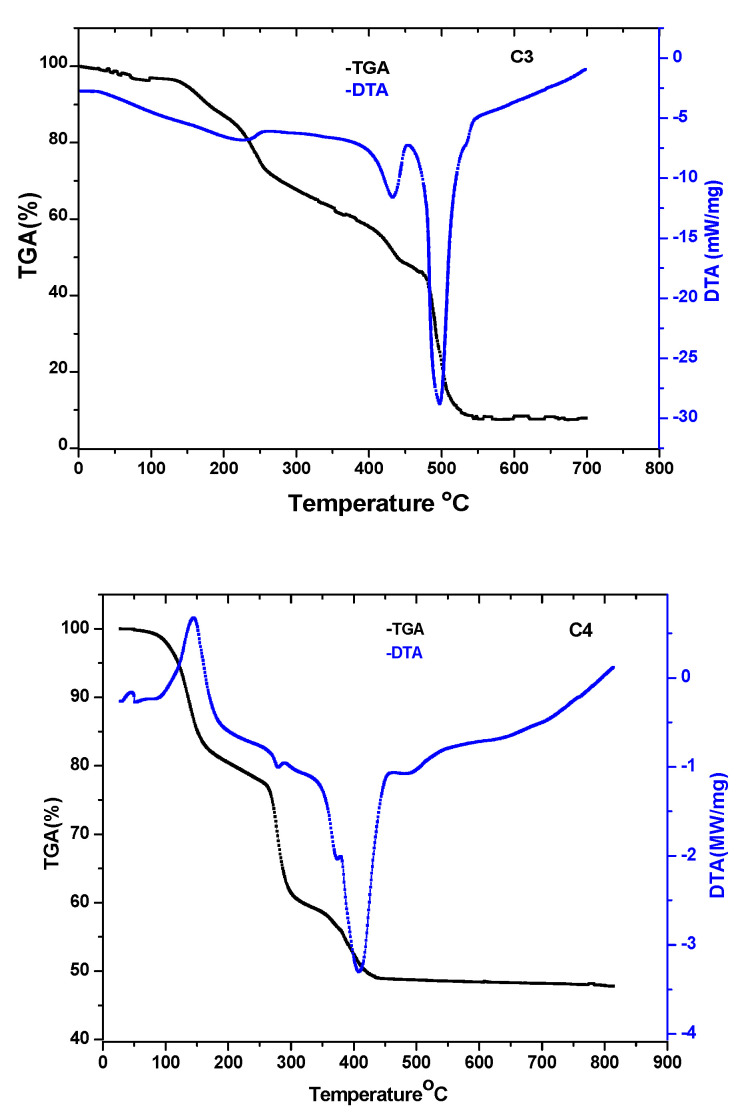
TGA and DTA of **C3** (**top**) and **C4** (**bottom**).

All the complexes under study were investigated on a thermograph over a wide range of temperature, from 27 to 800 °C, under inert atmosphere at a heating rate of 10 °C/min. The thermal behavior of the complexes **C1** and **C3** were similar, where they undergo decomposition in three steps [40]. In Figure 2, decomposition of **C3** is shown, in which the initially weight loss of 8.4% (calc. 8.2%) corresponds to the departure of the chloride molecule and which occurred in the range of 130–210 °C. The next weight loss began at 270 °C and ended at 390 °C due to the loss of two molecules of 5-fluorosalicyladihyde and accounted for 38.96% (calc. 37.12%). A major weight loss of 45.43% (calc. 45.45%) occurred in the range of 440–490 °C due to the departure of two molecules of aminomethylbenzimidazole. The final product was left behind as Co_2_O_3_. 

The thermograms of **C2** and **C4** showed a similar pattern of degradation. The four deflections in the thermogram indicate the thermal instability of the complexes. During the early stages of the degradation process, lattice water and chloride get eliminated over a temperature range of 78–230 °C with a loss of 8.91% (calcd. 8.91%). A major weight loss of 30.43% (calc. 30.23%) was attributed to the elimination of 1,10-phenantholine around 265–330 °C. The third deflection in the thermogram, due to the degradation of 5-fluorosalicyladihyde, appeared at 390–470 °C, with a mass loss of 19.35% (20.16%). Finally, aminomethylbenzimidazole left, with a weight loss of 24.35% (calcd. 24.69%) over the range 490–520 °C. Above 540 °C, a constant thermal stability indicates the formation of respective metal oxides. 

#### 3.1.4. Measurements of Molar Conductance and Magnetic Moment

Millimolar solutions of all the prepared metal complexes (**C1**-0.599, **C2**-0.615, **C3**-0.595, and **C4**-0.629 mm) were prepared using DMSO solvent and the conductance was measured (Table S1). Measurement of conductance at different intervals of time in solution showed a constant conductance, which reveals the stability of all the complexes in solution. The presence of chloride ion outside the coordination sphere was confirmed by huge conductance in the complexes **C3** and **C4** in comparison with the complexes **C1** and **C2,** which do not contain chloride ions outside the coordination sphere. The molar conductance of the Co(III) ion as well as the Cu(II) ion shows 134 µS and 154 µS, respectively, at 25 °C. The decrease in conductance was attributed to the formation of the complexes (**C1**–**C4**). 

The magnetic moment of the Cu(II) complex was observed in the range of 1.87–1.89 BM. This indicates that the existence of an octahedral environment around the complex molecular system. Further, the complex containing the Cu(II) ion is paramagnetic in nature. The Co(III) complexes are diamagnetic in nature. The presence and absence of the magnetic moment reveals valuable information regarding the geometry of the obtained complex, that is the Cu(II) ion fits in the octahedral geometry because of the effective magnetic moment and the Co(III) shows a d^6^ system, which is a low spin complex and correlated to octahedral geometry [41].

#### 3.1.5. Mass Spectra

Mass spectra of all the complexes are shown in (Figures S16–S19). The mass spectrum of **C1** showed a [M + 1]^+^ peak at *m*/*z* = 600.1157, which is in agreement with the theoretical value (600.0774). Similarly, **C2** exhibited peaks at *m*/*z* = 600.1157 (M^+^) (theoretical *m*/*z* = 601.51590) which suggests a hydrated complex in good agreement with the IR spectra [42]. The molecular [M]^+^ ion peak of the complex **C3** in the mass spectra could be seen at *m*/*z* = 630.48 and the [M-Cl]^+^ ion at *m*/*z* = 595.46. The mass spectrum of **C4** registered the molecular ion peaks at *m*/*z* = 614.1064 (theoretical value *m*/*z* = 614.34082) due to the mixed ligand complex formed by the co-ordination of planar moiety 1,10-phenothroline.

#### 3.1.6. ESR Spectra

**Figure 3:**
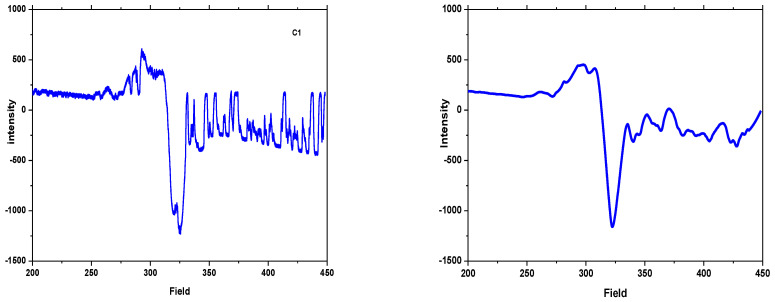
ESR Spectra of C1 (**right**) and C2 (**left**) complexes.

The paramagnetic behavior of the Cu(II) ion was studied by ESR spectral analysis. The ESR spectra of the Cu(II) complexes, **C1** and **C2** were measured on the X-band at liquid nitrogen temperature in DMSO (Figure 3). In the case of **C2**, the ESR spectra showed that the coupling interaction of the unpaired electron with the copper nuclei were too weak, so it appears to be a single main band with several small peaks and it should be less than **C1**, which indicates that the unpaired electron strongly polarized into the oxygen atom. Whereas in the case of **C1**, in addition to a main single band, there were several small peaks observed on either side, indicating the delocalization of an unpaired electron into the nitrogen atom of the benzimidazole moiety and azomethine group via a super-hyperfine coupling interaction [43]. The complexes, **C1** and **C2** showed g_||_ and g_⊥_ values in the region 2.343–2.191 and 2.070–2.066, respectively (Table 1). The value of g_||_ and g_⊥_ strongly suggests the distorted octahedral coordination of the Cu(II) ion and also because of the characteristic feature of axial symmetry, the unpaired electron predominantly in d_x_^2^__ y_^2^ orbital of Cu(II) ion (value of g_⊥_ was greater than 2.0023 and less than g_||_). The parameters of exchange coupling value were derived by the expression G = g_||_ − 2.0023/ g_⊥_ − 2.0023 [44].

### 3.2. Biology

#### 3.2.1. Pharmacology

##### Cytotoxicity and Structural-Activity Relationship

**Figure 4:**
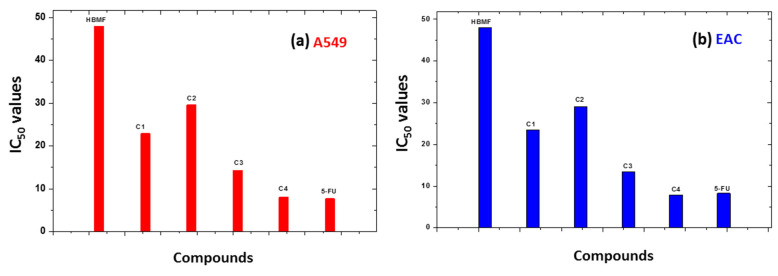

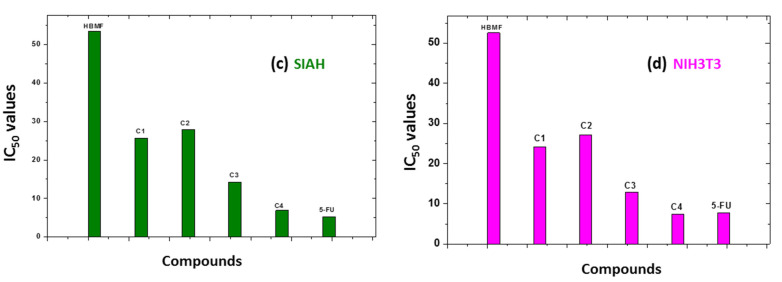
Screening of **HMBF** and its complexes (**C1**–**C4**) for their cytotoxicity and anti-proliferative potency against (**a**) A549, (**b**) EAC, (**c**) SIAH and (**d**) NIH3T3 depicting their respective IC_50_ values.

Metals wrapped with benzimidazole-based moieties govern profound interest in tailoring an anticancer pharmacophore. Recent studies suggest that benzimidazole salen complexes show potent anti-proliferative activity on various cancer cell lines [45,46]. The present investigation on the cytotoxicity and anti-proliferative properties of the novel coordinated benzimidazole derivative and phenanthroline scaffolds, were assessed against an EAC cell line in vitro as a primary screening. It is noteworthy that out of all the tested compounds, the complex **C4**, bearing coordinated benzimidazole derivative with fluoro substitution along with 1,10-phenanthroline as an axial ligand, exhibited significant cytotoxicity and proliferation inhibiting potency on the EAC cancer cell line with an IC_50_ value 10 µM in the MTT assay [47]. Cellular multiplication was determined by the MTT assay following treatment of the EAC cells with all the complexes (0–100 µM), which confirms that the complex **C4** with IC_50_ values of 10 µM was the most active drug-like molecule. Further, the complex **C4** was subjected to in vivo proliferation inhibiting investigations (Figure 4). 

##### Hematological and Serum Profile Parameters of Cobalt Complex

Significant changes were observed in the hematological parameters after the 20th day of treatment on the EAC cell bearing mice when treated with the complex **C4** at maximum dose of 10 mg/kg as compared with the EAC control group. The total WBC count was drastically increased in the EAC control cells, whereas alkaline phosphatase and urea values decreased in EAC bearing groups. The aforesaid parameters either increased, or decreased, from the normal value when treated with the complex **C4** and restored to normal value after the 25th day of the treatment. In comparison with the control EAC carrying mice, an about 48.36% reduction was observed in the number of EAC cells when treated with **C4** at a dose of 10 mg/kg (Table 2). At a higher dose of **C4** in normal mice, there is a hike in the number of macrophages and peritoneal cell to some extent (Tables S2–S4). 

##### Complex C4 Inhibits EAC Tumor Cell Growth

Cellular apoptosis evasion, uncontrolled growth and angiogenesis are the main hallmarks of cancer disease irrespective of their origin. The inhibition of these hallmarks is one of the main functions of an anticancer drug [48]. The introduction of the EAC cell line into normal mice induced a local inflammatory reaction which enhanced the vascular permeability followed by cellular migration and significant ascite fluid formation. The cancerous cells depend on the ascite fluid, which contains essential nutrients for its growth. Hence, suppressing the ascite fluid formation rapidly inhibits the tumor growth [49,50,51].

**Figure 5:**
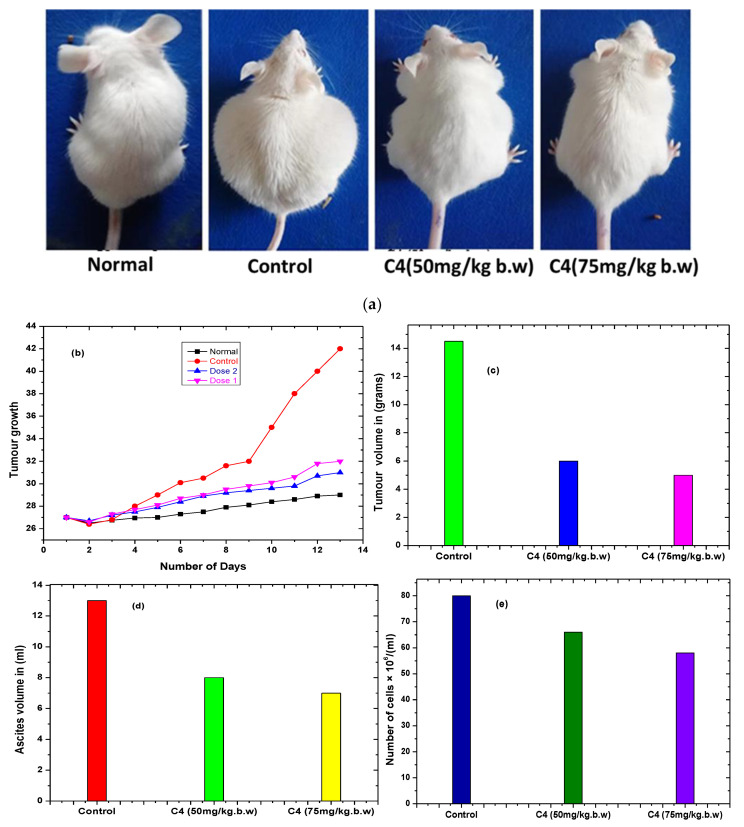

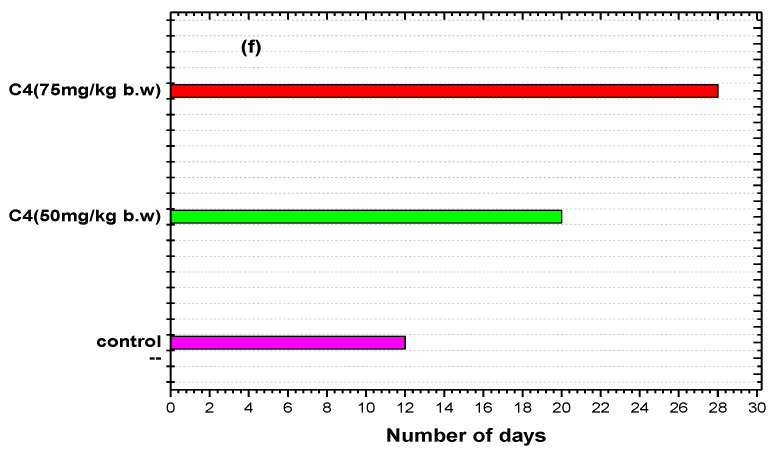
Represents the effect of complex **C4** on Ehrlich Ascites Carcinoma (EAC) in mice. The EAC cells intraperitoneal injected induced an ascite tumor in one and half month-old Swiss albino mice. Two doses of complex **C4** (50 mg/kg and 75 mg/kg) were each administered to the tumor-bearing mice on alternate days after three days of tumor growth. (**a**) Morphology of tumor bearing mice. (**b**) Repressed tumor volume. (**c**) Repressed tumor volume in gram. (**d**) Decrease in ascites secretion. (**e**) Reduction in tumor cell proliferations were analyzed by control versus treated. (**f**) Kaplan–Meier showing the extended survivability.

In the present investigation, the active scaffold, complex **C4**, was administered intraperitonially (i.p.) at a dose of 50 and 75 mg per kg body weight as assessed by LD50 studies. The complex **C4** shows significant tumor inhibiting activity and reduction in tumor growth after the 5th day of the administration, as evident from the physical morphology and tumor volume depicted in Figure 5a–c. The tumor inhibiting activity increases with the increase in dose as per the pharmacological studies. The results also suggest that there was suppression in the ascite fluid formation and tumor cell counts as shown in Figure 5d, e. In comparison with the control, the survivability of treated mice was increased to two-fold higher from the 12th day to the 26th day (Figure 5f) [52]. The acute toxicity of the lead complex was also investigated, which indicates that the internal organs were almost normal in comparison to the tumor treated mice. The untreated mice showed an enlarged spleen and liver, which confirms the potency of the complex **C4** in anti-proliferative action.

##### Angiogenesis

**Figure 6:**
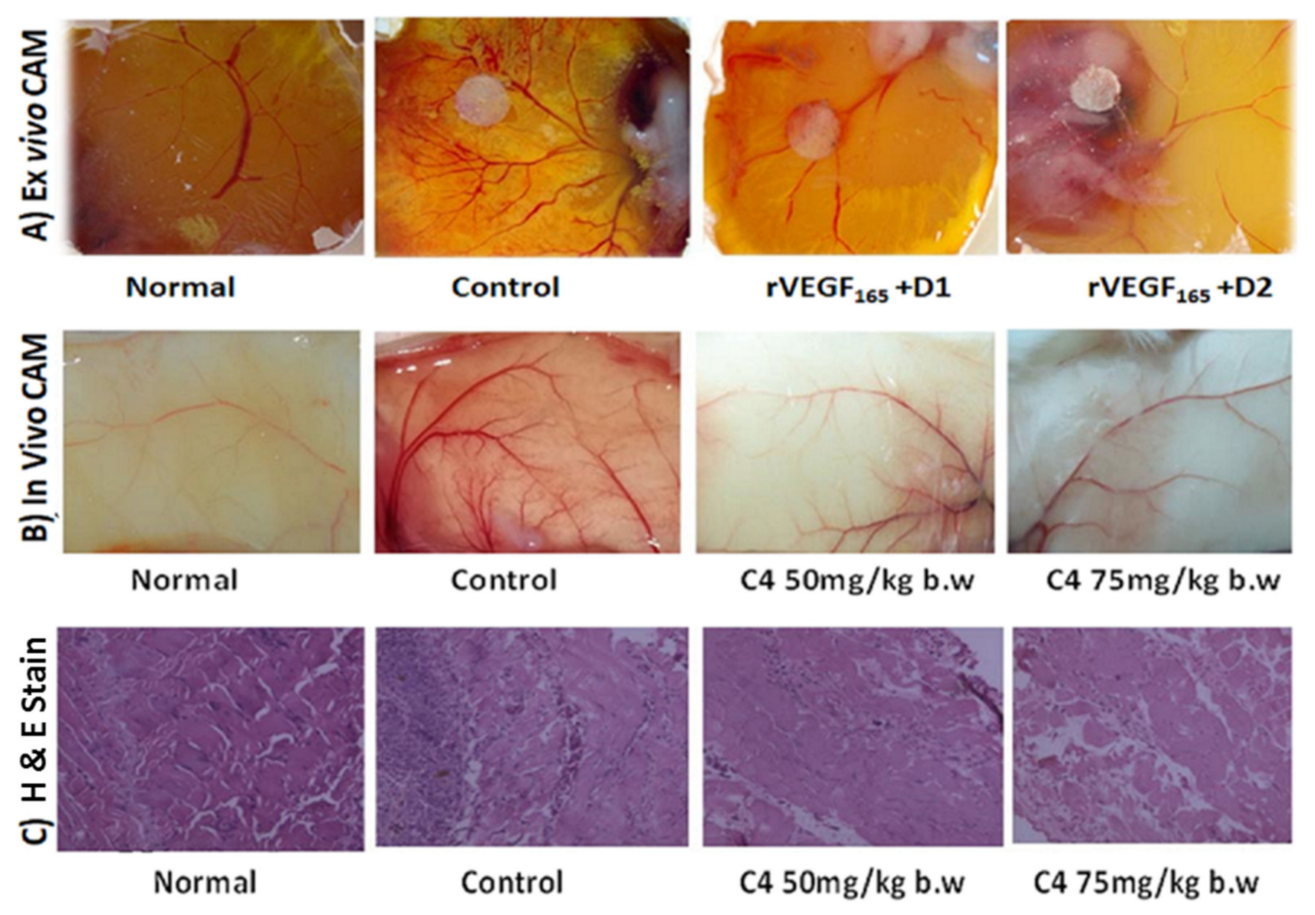
Complex **C4** antagonizes the microvascular formation in non-tumor antigenic models. The effect of complex **C4** on neovascularization was studied by stimulating angiogenesis with rVEGF_165_ and treating with the complex **C4** the non-tumor models. (**A**) Ex vivo and (**B**) In vivo CAM Photographs represent the inhibition of neovasculature formation, (**C**) Antiagiogenic effect of complex **C4** was evident from haematoxylin and eosin staining of peritoneum section showing the decreased in vascular counts.

Cellular apoptosis at a slower rate and angiogenesis at a faster rate have direct consequences on cell proliferation. Angiogenesis is a normal and complicated process involving reconstruction of blood vessels from existing blood vessels [53]. Neovascularization has a prominent significance in wound repair, embryonic development, and inflammation controlled mainly by VEGF (Vascular epidermal growth factor). Over-activation of VEGF accelerates angiogenesis which recruits various inflammatory cells by signaling through pro-antigenic molecules and that results in the formation of a tumor. In order to suppress tumor growth, one of the ways is to arrest angiogenesis by blocking VEGF. The blocking of angiogenesis promotes cellular apoptosis resulting in cell death, and thereby hampers tumor growth [54]. Angiogenesis controls the tumor growth and metastasis, as exploited in many in vivo assays. Increase in tumor growth and hematogenous tumor embolization is mainly attributed to enhanced neovasculature. Therefore, hindering tumor angiogenesis may arrest tumor growth and depress metastatic potential of tumors. Many researchers have suggested that elevated vascularized tumors confer a low rate of survival, whereas depressed vascularized tumors are associated with a high rate of survival. Measurement of the extent of neovascularization or micro vessel density is widely used as a surrogate marker in pathological specimens and tumor models to assess the prognosis of the disease. Therefore, the EAC cell line, which is a convenient model to study the MVD, was used in this experimentation. The important modes of angiogenic assays are CAM and H&E staining examination were carried out to assess the effect of **C4** in MVD. The results showed that the compound **C4** significantly reduces the vascular supply as appeared in peritoneal angiogenesis and the CAM assay (Figure 6A,B). Hence the depression of neovascularization by C4 observed in H&E staining provides strong evidence for the compound’s anti-angiogenesis property (Figure 6C).

### 3.3. Computational Results 

#### 3.3.1. Local Reactivity of Ligand

Local reactivity properties of the studied molecular structures were addressed by considering the topology and distribution of the frontier molecular orbitals (FMO) and the selected quantum-molecular descriptors. The DFT calculations at the M06-2X/LACVP(d,p) level of theory were applied to obtain this information. The FMOs are the most critical molecular orbitals from the aspect of local reactivity. Molecular sites hosting the highest occupied molecular orbital (HOMO) are rich with electrons, while the location of the lowest unoccupied molecular orbital (LUMO) shows the molecular sites that could host electrons. The energy difference between these orbitals is an indicator of stability, while the locations of these orbitals provide information about the charge transfer upon excitations. These two descriptors, probably the best-known and the most frequently applied descriptors of local reactivity, are suitable for identifying the most reactive sites of molecules. Combining these two quantum-molecular descriptors is highly recommended to obtain a clearer reactive picture of the studied molecular structures because they indicate two types of sensitivity [54]. 

**Figure 7:**
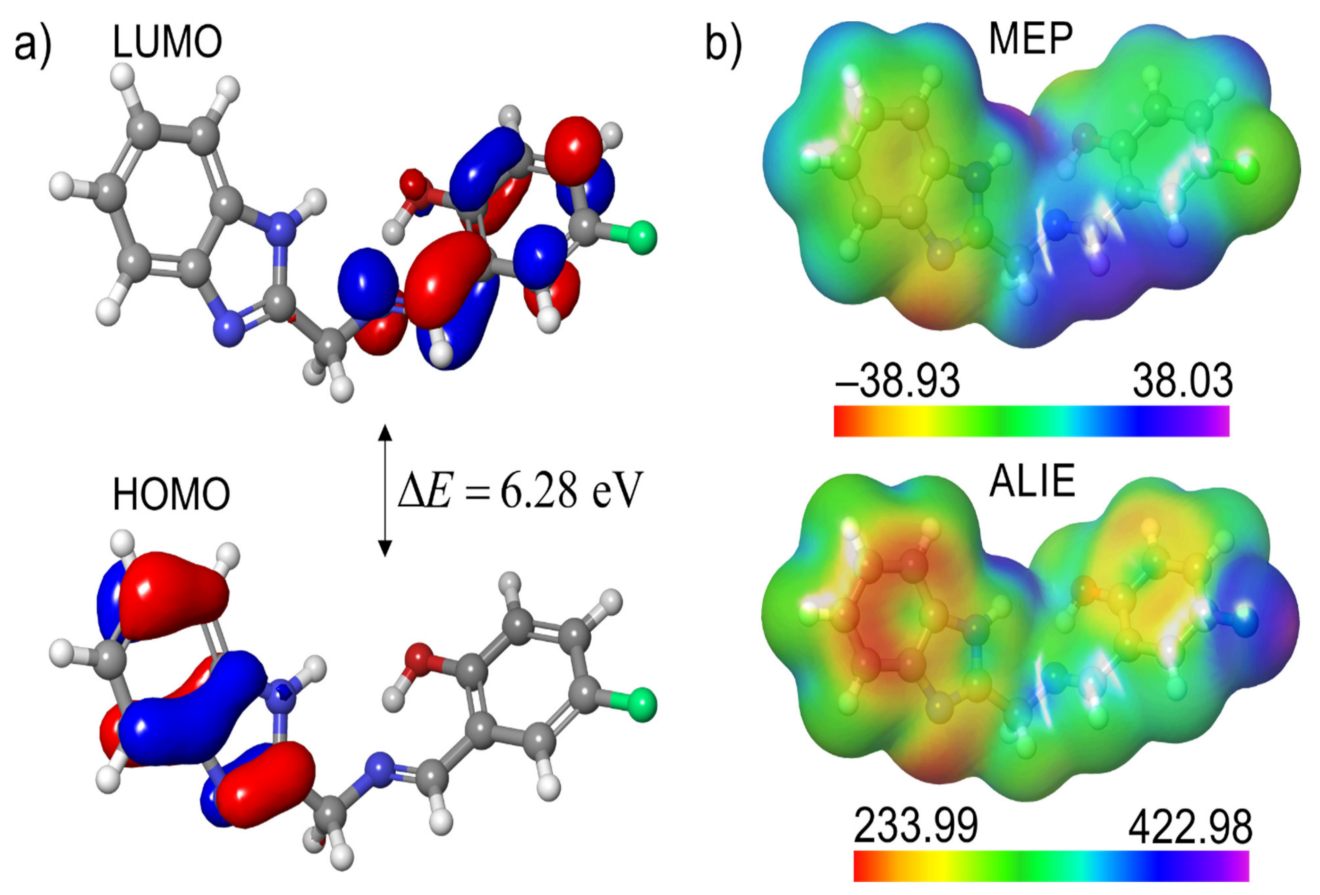
(**a**) FMOs and (**b**) MEP and ALIE surfaces (with minimal and maximal values) of the HBMF molecule. Maximal and minimal MEP and ALIE values are given in kcal/mol. Surfaces have been obtained by taking the isovalues of ±0.050 a.u. and ±0.001 a.u. for FMOs and MEP/ALIE, respectively.

The strength of MEP is that it identifies the molecular sites sensitive to react with other molecules based on noncovalent interactions. On the other hand, ALIE is suitable for identifying the molecular areas susceptible to electrophilic attacks. These descriptors were presented in the form of surfaces by mapping their values to the electron density surface. In Figure 7, we show the FMOs, MEP, and ALIE surfaces of the HBMF molecule.

The FMOs presented in Figure 7a indicate that HOMO and LUMO of the HBMF molecule are well separated. The HOMO is delocalized over the molecular area where benzene is fused with an imidazole fragment. This molecular area is rich in electrons and can donate charge while interacting with other species. On the other hand, LUMO is delocalized over the molecular area containing the other benzene ring and bridging chain. This molecular area is electron-defiant and is prone to accept electrons from other species [55,56]. Due to the large separation of FMOs, significant charge transfer from one part of the molecule to another occurs due to the excitation. The energy difference between HOMO and LUMO is also quite substantial (6.28 eV), indicating high stability of the HBMF molecule.

The MEP surface presented in Figure 7b reveals two characteristic reactive centers of HBMF. Near the vicinity of the nitrogen atom of the imidazole fragment is characterized by the lowest MEP values (−38.93 kcal/mol), indicating that this part of the molecule is susceptible to interact with positively charged fragments of other molecules. On the other hand, near the vicinity of the hydrogen atom connected to another nitrogen atom of the imidazole fragment is characterized by the highest MEP values (38.03 kcal/mol), indicating that this part of the molecule is susceptible to interaction with negatively charged fragments of other molecules. The ALIE surface presented in Figure 7b recognizes these two characteristic molecular sites as the lowest and highest ALIE values are also located there. According to the ALIE descriptor definition, imidazole’s nitrogen atom is the most susceptible to electrophilic attacks. Additionally, the lowest ALIE values are also located near the benzene ring that is fused to the imidazole ring, indicating that this part of the molecule might also be susceptible to electrophilic attacks.

#### 3.3.2. Interactions of HBMF with Water

**Figure 8:**
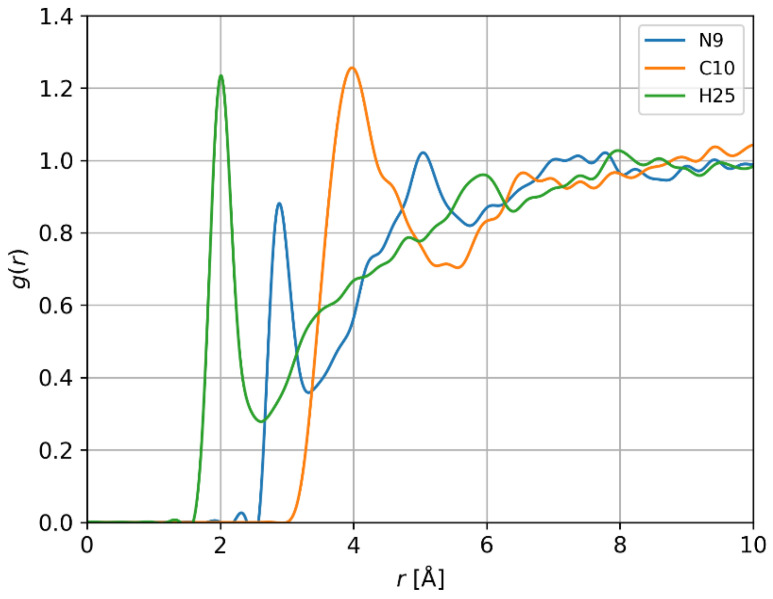
Representative RDFs of the **HBMF** molecule.

Knowing how the ligand interacts with water is important for understanding their properties in biological systems [55]. For these purposes, we performed a forcefield-based MD simulation of the HBMF molecule surrounded by approximately 2000 water molecules. After obtaining the respective trajectories for the simulation time equal to 10 ns, we performed calculations of radial distribution functions, aiming to identify the most reactive atoms towards the water molecules. The RDFs were calculated with respect to the distance between the atom of the HBMF and the oxygen atom of the water molecules. Representative RDFs, in terms of g(r) shape, are presented in Figure 8. 

**Figure 9:**
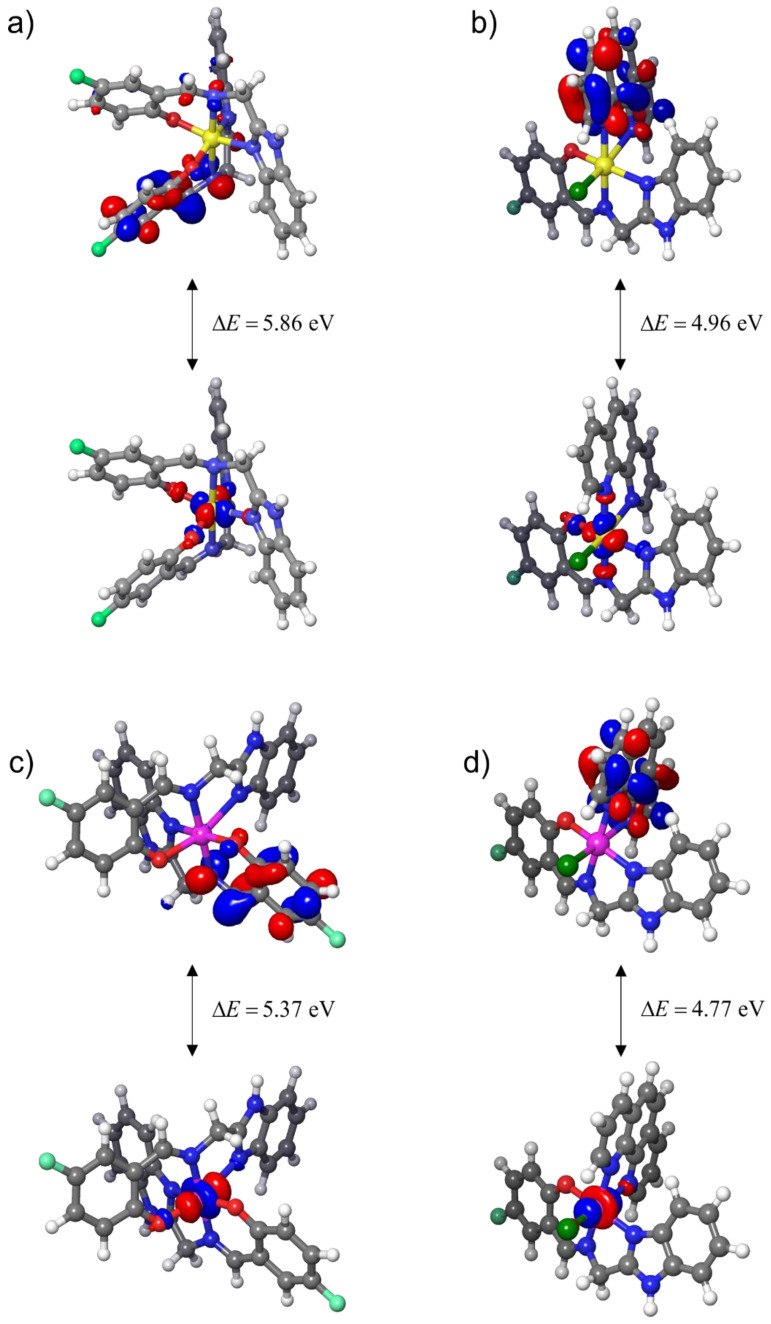
The FMOs of studied complexes (**a**) C1, (**b**) C2, (**c**) C3 and (**d**) C4 with corresponding HOMO-LUMO gap values ΔE Surfaces were obtained by taking the isovalue of ±0.050 a.u.

The RDF results presented in Figure 9 indicate that there is practically only one atom of the HBMF with pronounced interactions with water molecules. That is the hydrogen atom H25, the one connected to the nitrogen atom of the imidazole fragment. The highest MEP values characterize the same atom in Figure 7b. In particular, this atom was characterized by the sharp RDF whose maximal g(r) value was located at a distance of 2 Å, which indicates strong interactions with water molecules. The other two atoms of HBMF have relatively meaningful interactions with water molecules. Nitrogen atom N9 was characterized by the sharp RDF whose g(r) was located at a distance of around 3 Å. The relatively high g(r) value of carbon atom C10 is probably not significant as it was located at a distance of 4 Å.

#### 3.3.3. Local Reactivity of Complexes

**Figure 10:**
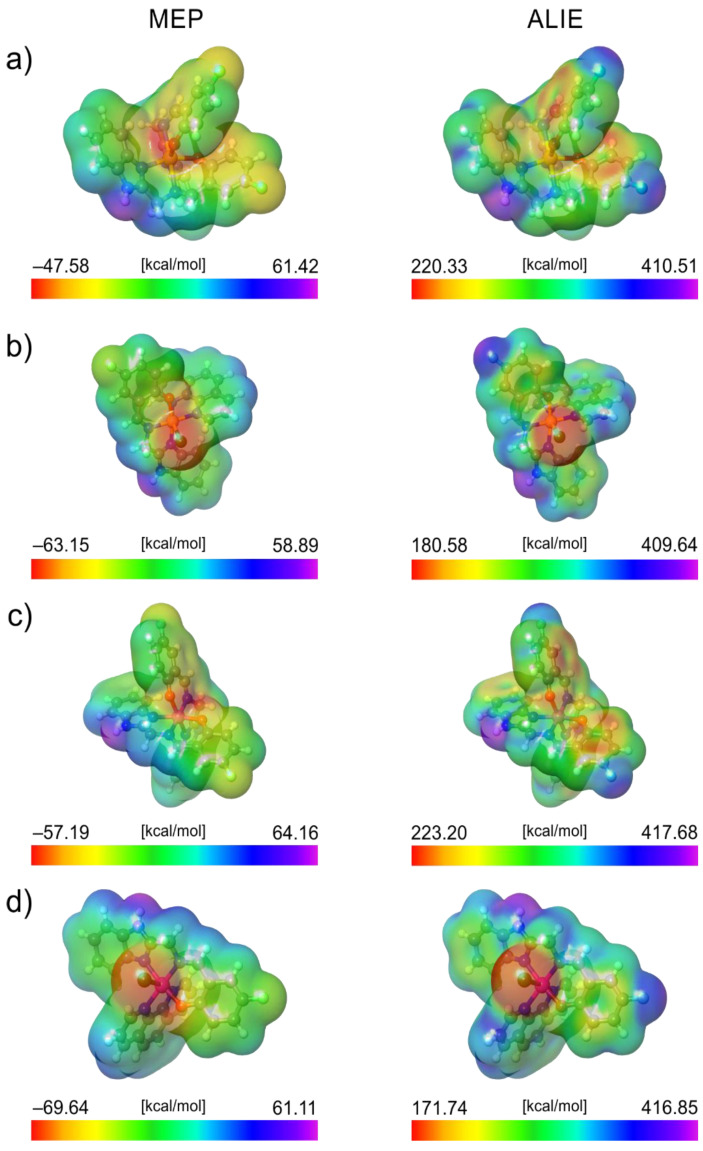
(**a**–**d**) MEP and ALIE values of studied complexes. Presented surfaces have been obtained by taking the isovalue of ±0.001 a.u.

**Figure 11:**
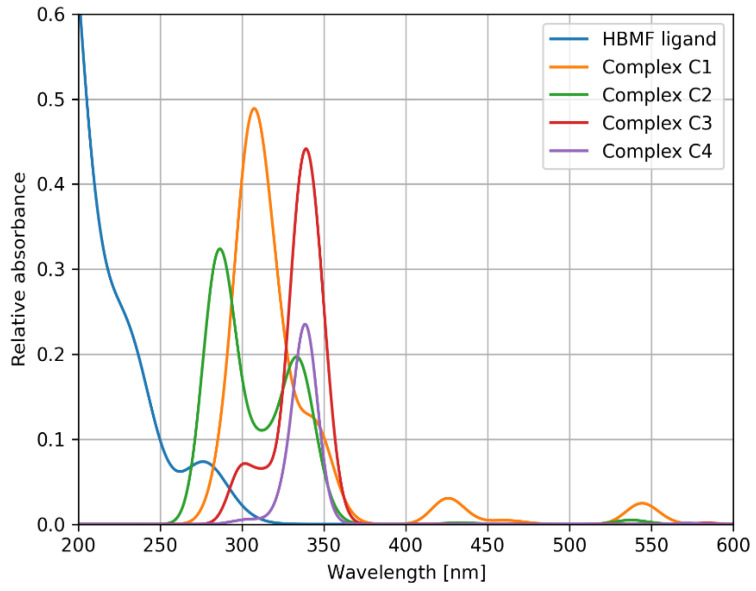
Simulated UV spectra of studied molecules.

Using the same level of theory, complexes were also subjected to single-point energy calculations to obtain information about their FMOs and the MEP and ALIE surfaces. The FMOs for the studied complexes are presented in Figure 10, while Figure 11 contains the respective MEP and ALIE surfaces.

Results related to the ΔE presented in Figure 9 indicate that the most stable complex is C1 with the corresponding value of 5.86 eV. Complex C1 is followed by C3 and C2 with corresponding values of 5.36 eV and 4.96 eV, respectively. The least stable complex according to the ΔE value appears to be the C4, with the corresponding value of 4.77 eV. In the cases of all complexes, the HOMO is delocalized over a small area including the metal, while the LUMO is always delocalized over a single fragment.

According to the MEP values, complex C4 appears to be the most reactive, in terms of the electrostatic interactions, towards positively charged species. Namely, this complex is characterized by the lowest MEP value of −69.64 kcal/mol, located near its chlorine atom (Figure 10). Regarding the lowest MEP values, C4 is followed by C2, C3 and C1, with corresponding values of −63.15 kcal/mol, −57.19 kcal/mol and −47.58 kcal/mol, respectively. Considering the electrostatic interactions, the most reactive complex towards the negatively charged species appears to be complex C3, whose highest MEP value is 64.16 kcal/mol. In terms of the highest MEP values, C3 is followed by C1, C4 and C2, with corresponding values of 61.42 kcal/mol, 61.11 kcal/mol and 58.89 kcal/mol, respectively.

The distribution of ALIE values is similar to MEP, with the additional presence of the lowest ALIE values above the benzene rings. However, there is a vast difference in the lowest ALIE values between the complexes, indicating a huge difference in susceptibility to electrophilic attacks. Namely, the lowest ALIE values of complexes C4 and C2 are 40–50 kcal/mol lower than the lowest ALIE values of complexes C1 and C3, indicating a huge difference in reactive properties. According to the lowest ALIE values, C4 again appears to be the most reactive complex.

#### 3.3.4. Simulation of UV Spectra

The TD-DFT calculations were used to analyze excitations and simulate the UV spectra for all studied structures. A long-range corrected version of the B3LYP functional, namely the CAM-B3LYP, was used to analyze excitations. Regarding the basis sets, the diffuse functions on heavy atoms were added, meaning that the employed basis set was LACVP+(d,p). Due to the size and complexity of the C1–C4 structures, excitations were analyzed in a Tam–Dancoff approximation. The simulated UV spectra are presented in Figure 11. 

The UV spectra presented in Figure 11 were obtained by broadening the oscillator strengths with the Gaussian function. The value of the half-bandwidth was set to 20. According to the experimentally obtained results, HBMF was characterized by the maximal absorption peak at around 300 nm. Our TD-DFT calculations were able to “capture” this absorption peak, however, it was located at a wavelength of about 275 nm. The matching between the experimental and computational UV spectra is outstanding in the cases of complexes C1 and C2. In both cases, there were practically two distinct absorption peaks, although there was a slight mismatch in the wavelengths of these peaks due to the approximation that had to be applied. The agreement between experiment and theory could be better in the cases of the complexes C3 and C4, in the terms that our TD-DFT calculations were unable to “capture” the second absorption peak at around 400 nm. However, one should consider that approximation was used, so overall, a good agreement was obtained. 

## 4. Conclusions

In summary, we have reported the synthesis and characterization of novel mononuclear benzimidazole saline complexes [Cu(HBMF)_2_], [Cu(HBMF)(phen)Cl]3H_2_O, [Co(BMF)_2_]Cl and [Co(BMF)(phen)Cl]Cl·2H_2_O by various instrumental analyses. The data are in good agreement with the proposed molecular structure, coordinating mode and molecular geometry. The complexes were thermally stable which was evident from TGA/DTA studies. Computational results in the case of the ligand indicated that the FMOs were well separated, with a high ΔE value denoting high stability. Considering the combination of MEP and ALIE descriptors, it appears that the highest reactivity might be expected for complex **C4**. The MD simulations identified hydrogen atom H25, belonging to the imidazole fragment, to have the strongest interaction with water molecules. Good agreement between experimental and simulated UV spectra was achieved. Furthermore, the pharmacological behavior of the metal complexes was studied by in vitro and in vivo anti-proliferative assay and anti-angiogenesis assay. From the in vitro and in vivo studies against EAC cell lines, the complex **C4** emerged as the lead compound with significant tumor suppressing properties with least toxicity. Moreover, complex **C4** accelerates apoptosis by slowing down neovascularization, which was evident from the anti-angiogenesis assay. One of the hallmarks of cellular apoptosis is nuclear fragmentation.

## Figures and Tables

**Table 1:** ESR spectral values (g_||_ and g_⊥_) and bonding parameters of **C1** and **C2** complexes.

Complexes	g_⊥_	g_||_	g_avg_	G	A_‖_	α^2^	f = g_‖_/A_‖_
**C1**	2.0243	2.0708	2.0398	3.5822	154	0.72	134
**C2**	2.0332	2.1591	2.0751	3.7510	171	0.67	126

g_⊥_—gyromagnetic ratio (perpendicular), g_||_—gyromagnetic ratio (parallel), g_avg_—gyromagnetic ratio (average), G—Lande splitting factor, A—Hyperfine coupling constant, α—Spin states (spin population).

**Table 2:** Showing hematological and serum profiles of the normal mice and normal non-tumor bearing mice treated with complex **C4**.

Hematological and Serum Profile Parameters	Normal Mice	Treated Mice
Alkaline Phosphatase (IU/L)	136.78 ± 1.30	134.45 ± 2.1
Creatinine (mg/dL)	0.39 ± 1.3	0.43 ± 1.25
Urea (mg/dL)	48 ± 2.2	43 ± 2.3
RBC (10^6^/μL)	5.7 ± 0.5	5.4 ± 0.6
WBC (10^6^/μL)	3.56 ± 2.3	3.5 ± 2.4

## Data Availability

The data presented in this study are available within the manuscript, or in the Supporting Information file.

## Supplementary Materials

The following supporting information can be downloaded at https://www.mdpi.com/article/10.3390/ph16010125/s1: Figures S1 and S2: proton and carbon NMR of the ligand; Figures S3–S8: UV-Visible spectra of the ligand and its complexes; Figures S9–S14: FT-IR spectra of the ligand and its complexes; Figures S15–S19: Mass spectra of the ligand and its complexes; Figures S20 and S21: Fragmentation pattern of the C2 and C4 complexes. Table S1: Molar conductance of complexes; Tables S2–S4: IC50 values of ligand and complexes.
