# Supplementary material for: Novel Benzimidazole Derived Imine Ligand and Its Co(III) and Cu(II) Complexes as Anticancer Agents: Chemical Synthesis, DFT Studies, In Vitro and In Vivo Biological Investigations
Source: Pharmaceuticals (Basel). 2023 Jan 13;16(1):125. doi: 10.3390/ph16010125 (PMC9866693; doi:10.3390/ph16010125)
Supplement: Supplementary file 1 [file pharmaceuticals-16-00125-s001.zip › pharmaceuticals-2078979-supplementary.pdf]

Supplementary Material

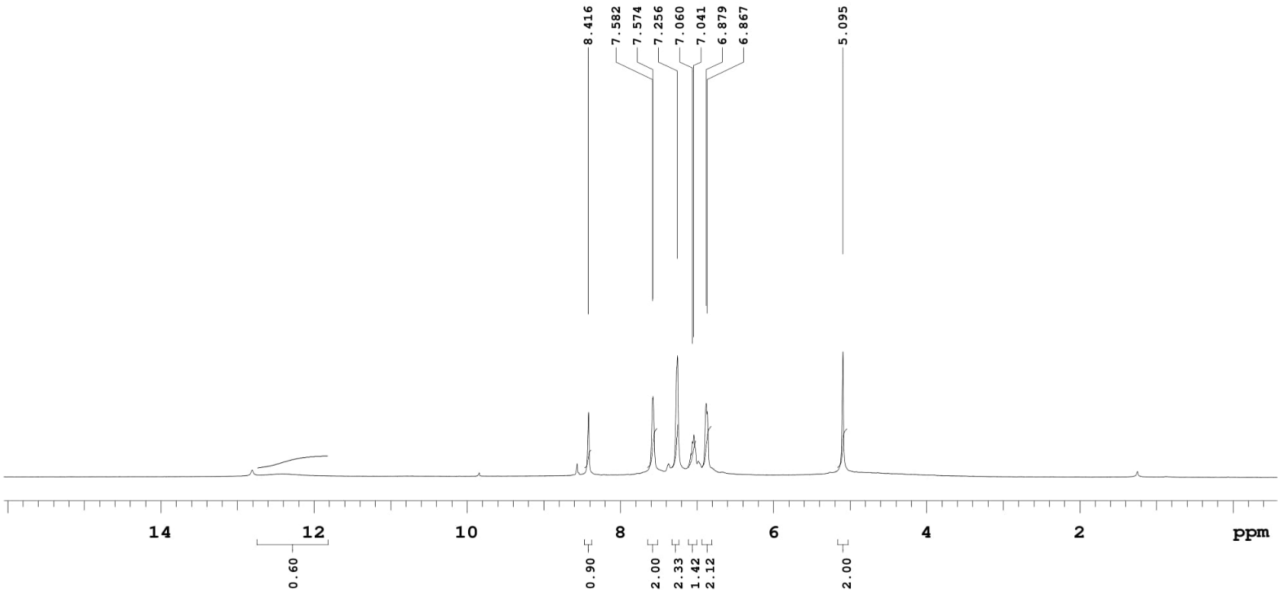

Figure S1: ^{1}H NMR spectra of ligand **HBMF**.

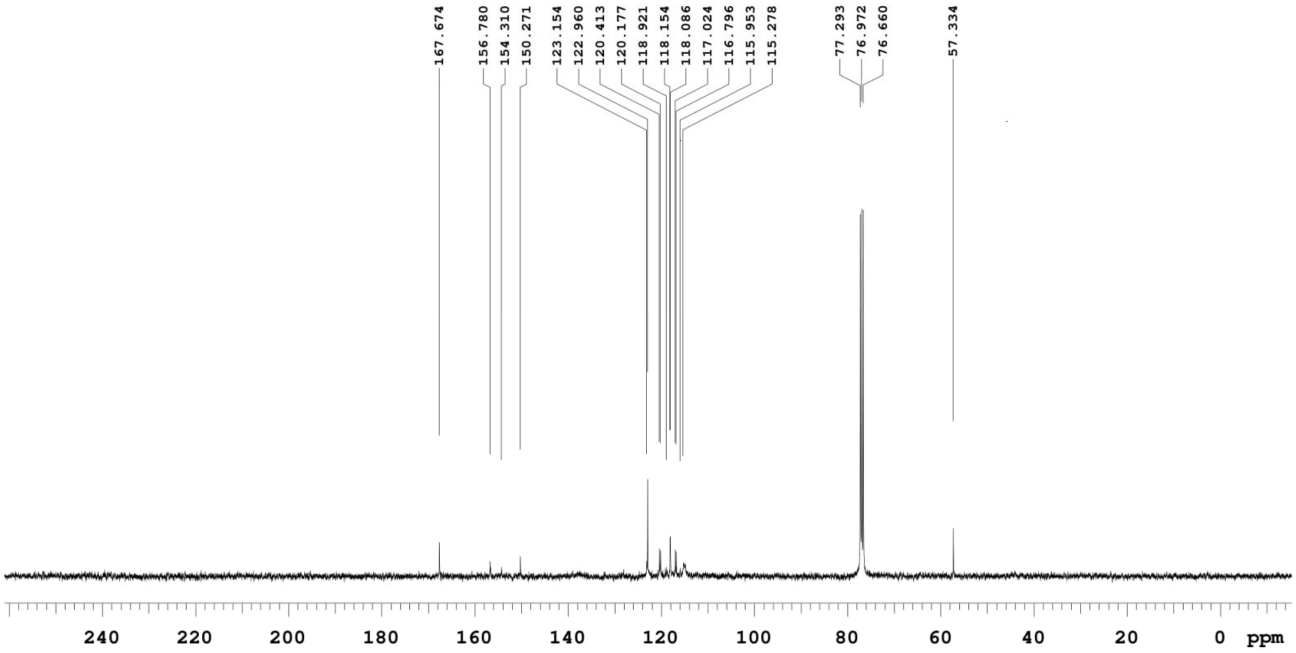

Figure S2: ^{13}C NMR spectra of ligand **HBMF**.

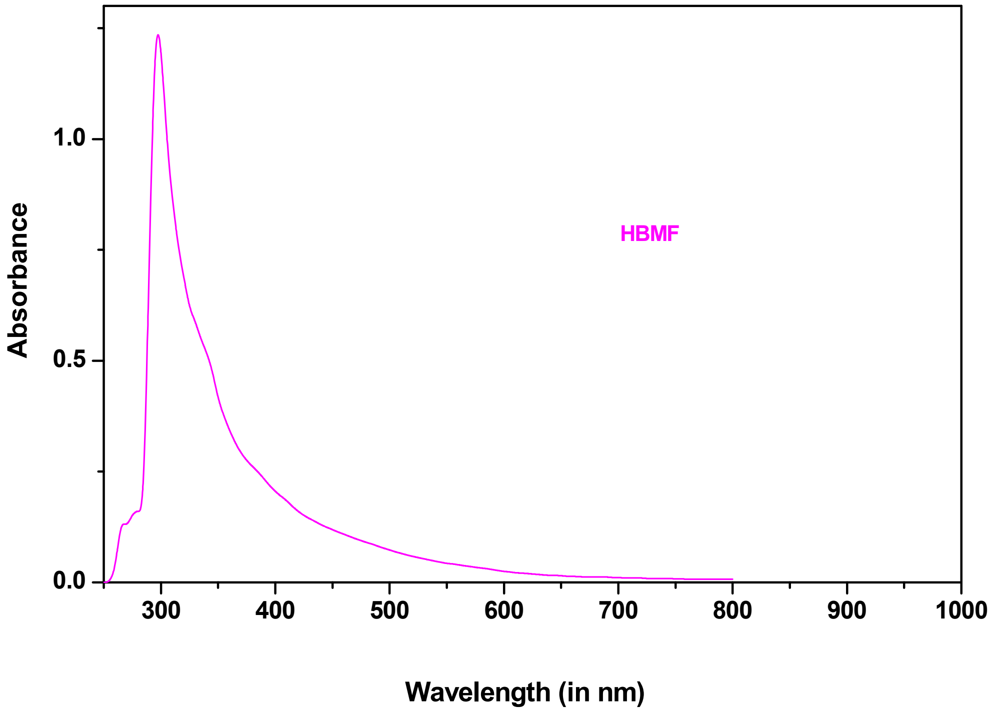

Figure S3. UV-visible spectrum of ligand **HBMF.**

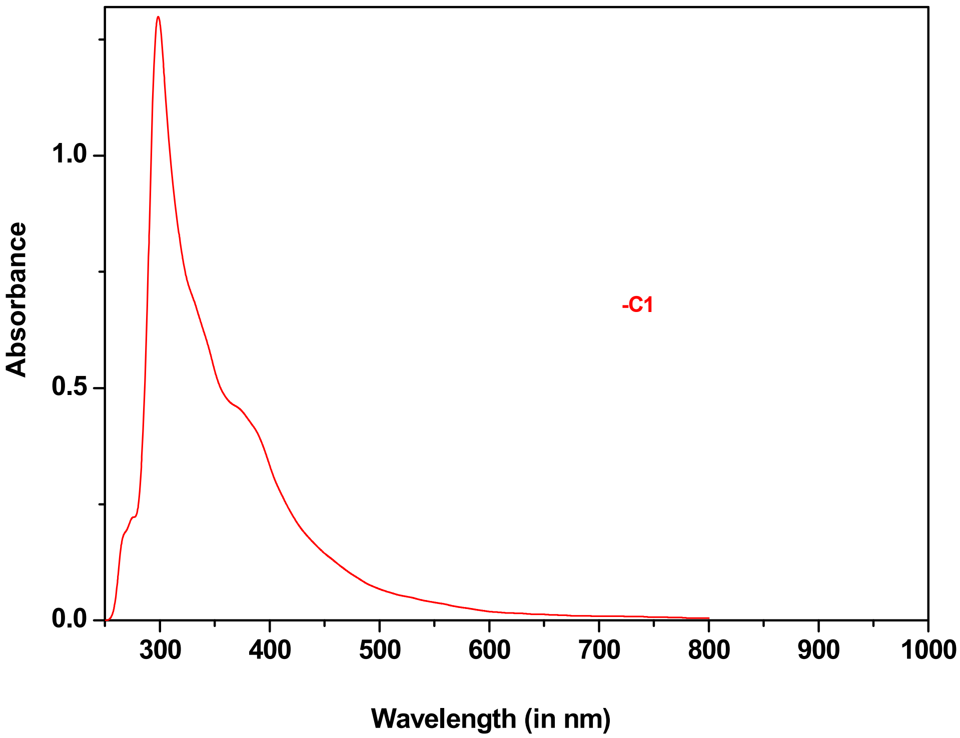

Figure S4. UV-visible spectrum of complex **C1.**

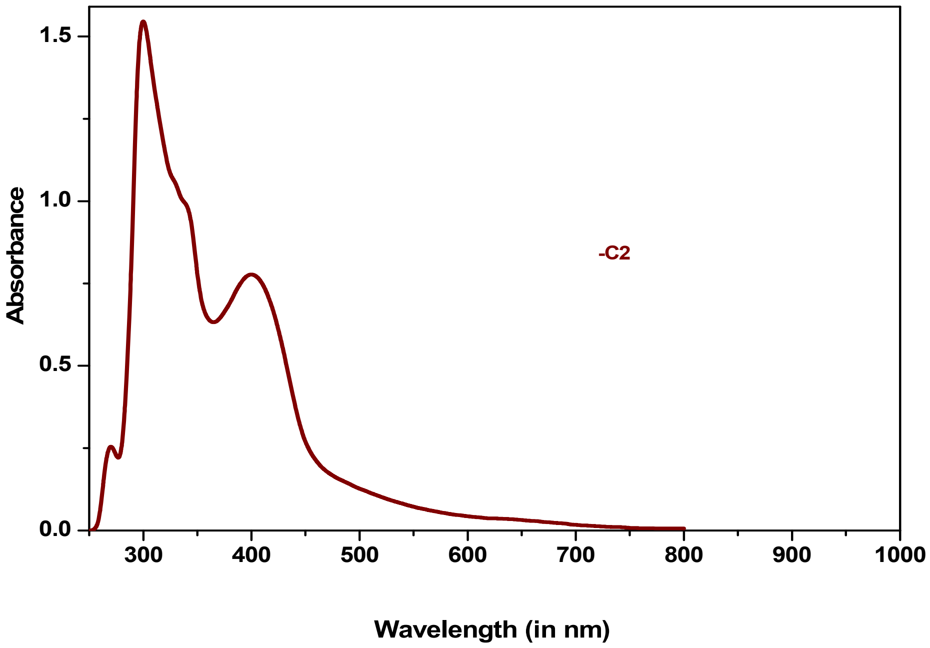

Figure S5. UV-visible spectrum of complex **C2.**

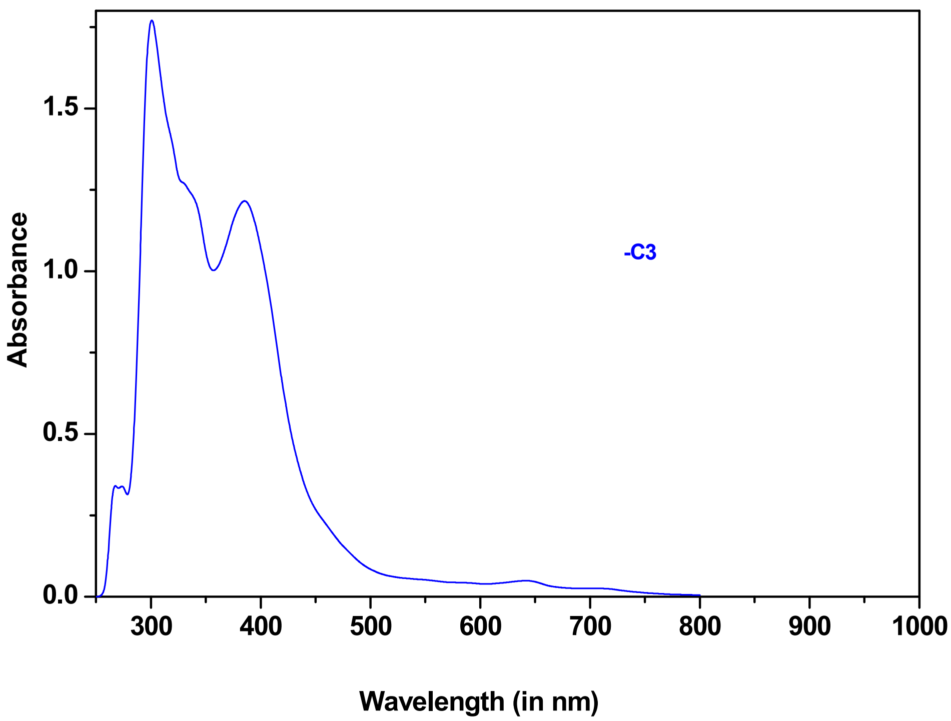

Figure S6. UV-visible spectrum of complex **C3.**

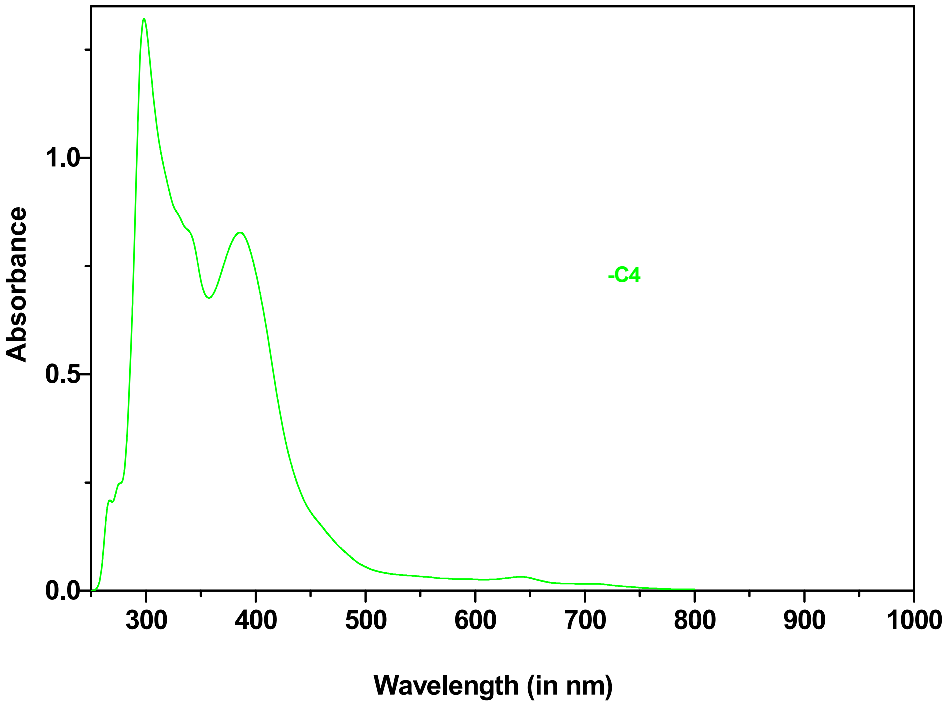

Figure S7. UV-visible spectrum of complex **C4**.

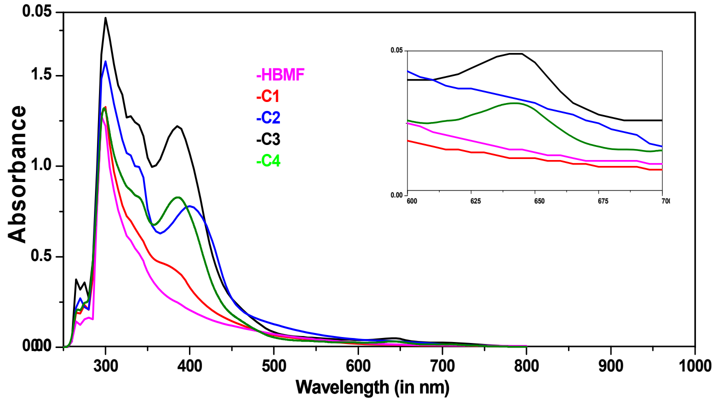

Figure S8. UV spectra of ligand (**HBMF**) and complexes (**C1, C2, C3** & **C4**).

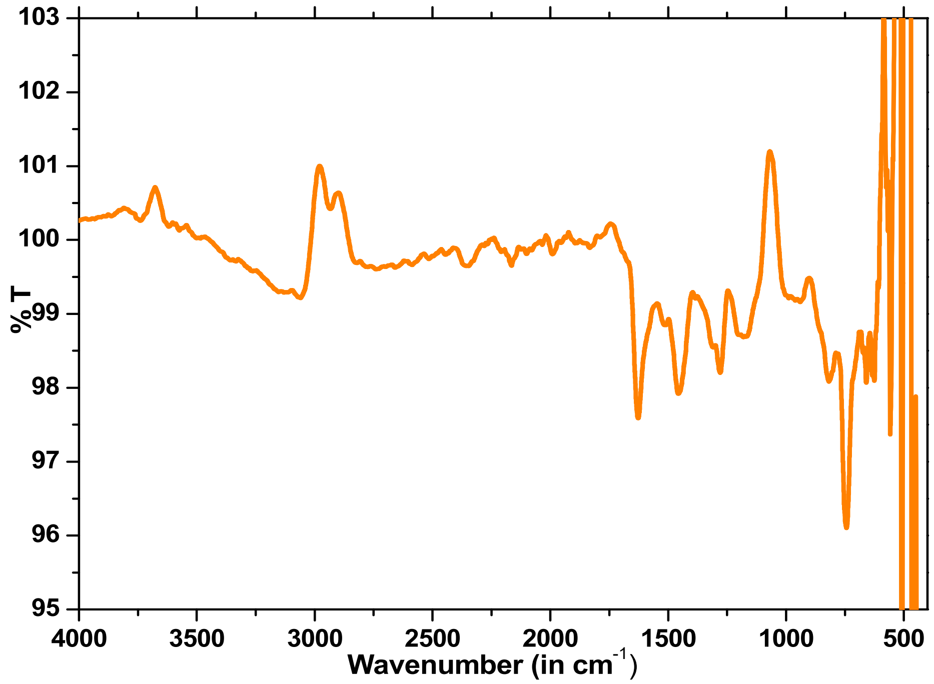

Figure S9. IR spectrum of ligand **HBMF.**

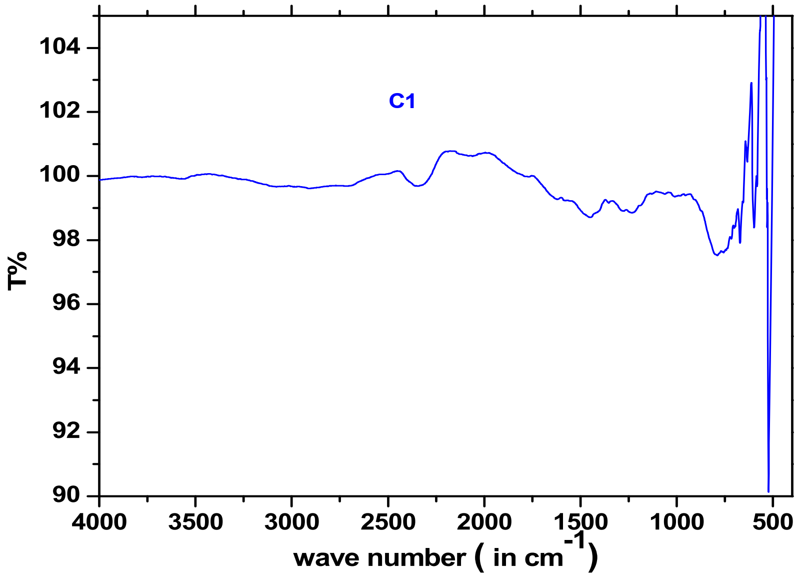

Figure S10. IR spectrum of complex **C1.**

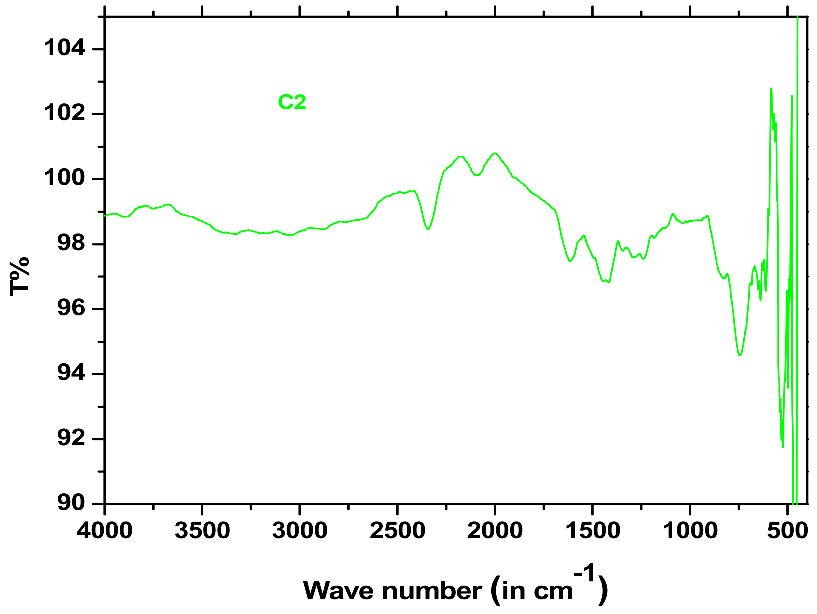

Figure S11. IR spectrum of complex **C2.**

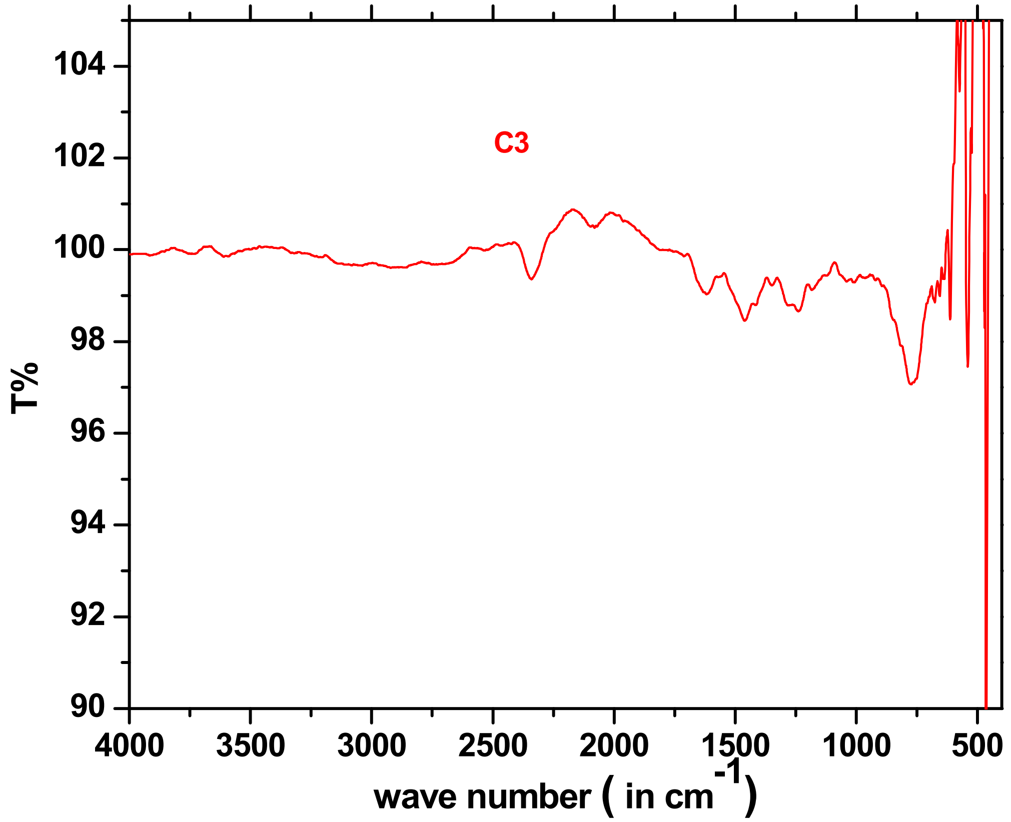

Figure S12. FT-IR spectrum of complex **C3.**

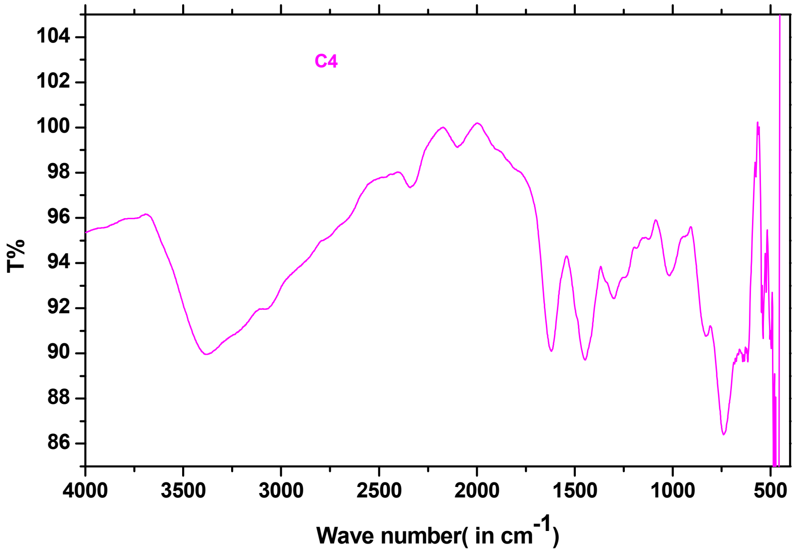

Figure S13. IR spectrum of complex **C4.**

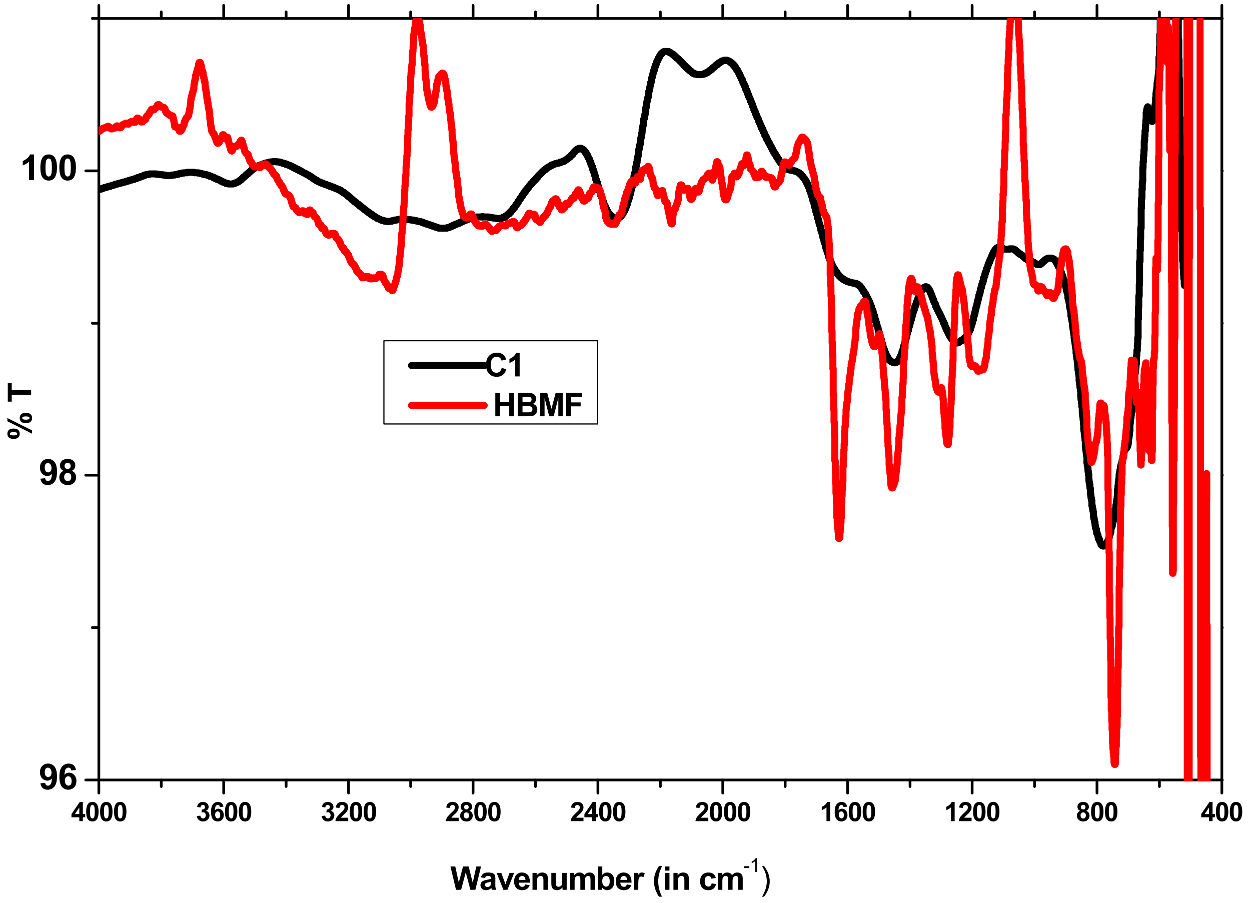

Figure S14. IR spectrum of ligand (**HBMF**) and complexes **C1.**

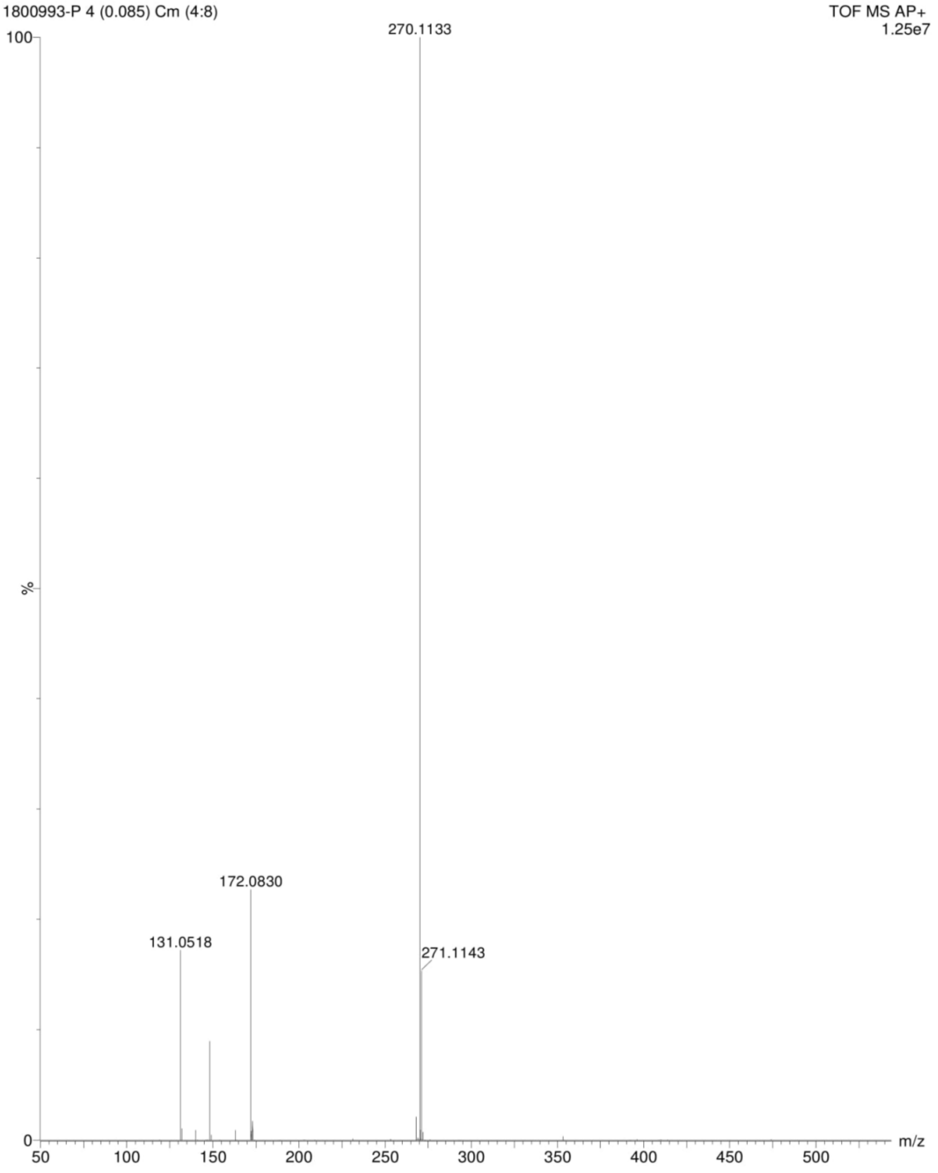

Figure S15: Mass spectrum of ligand **HBMF.**

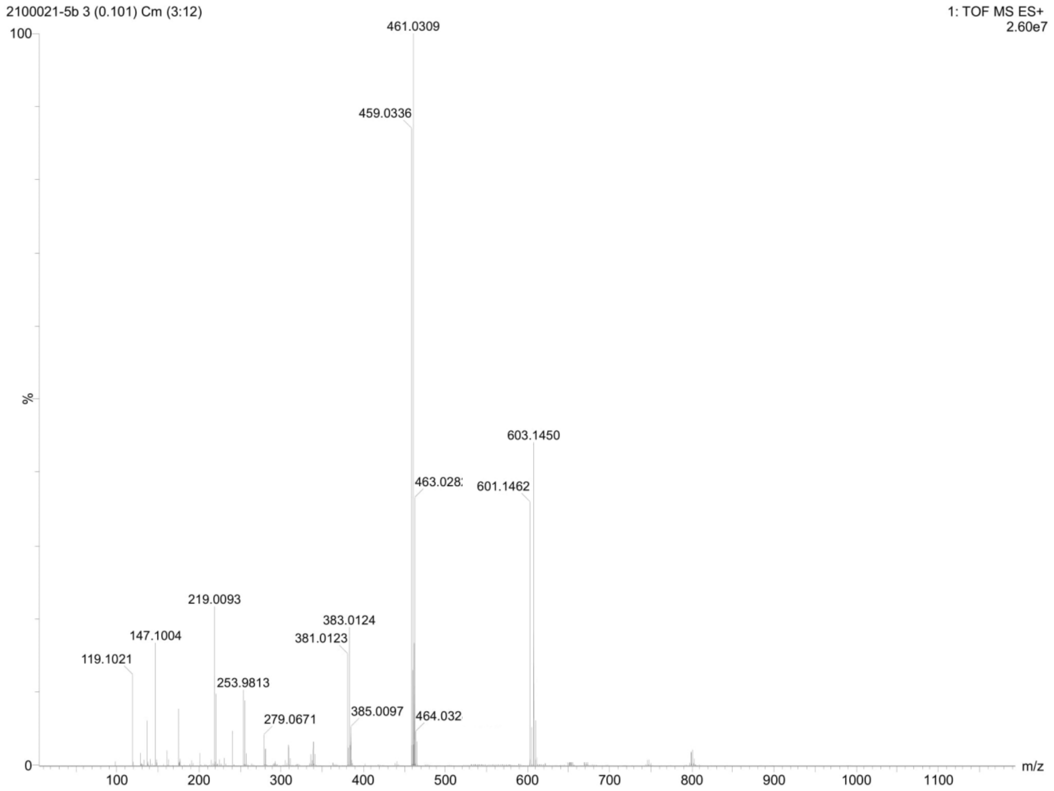

Figure S16: Mass spectrum of complex **C1.**

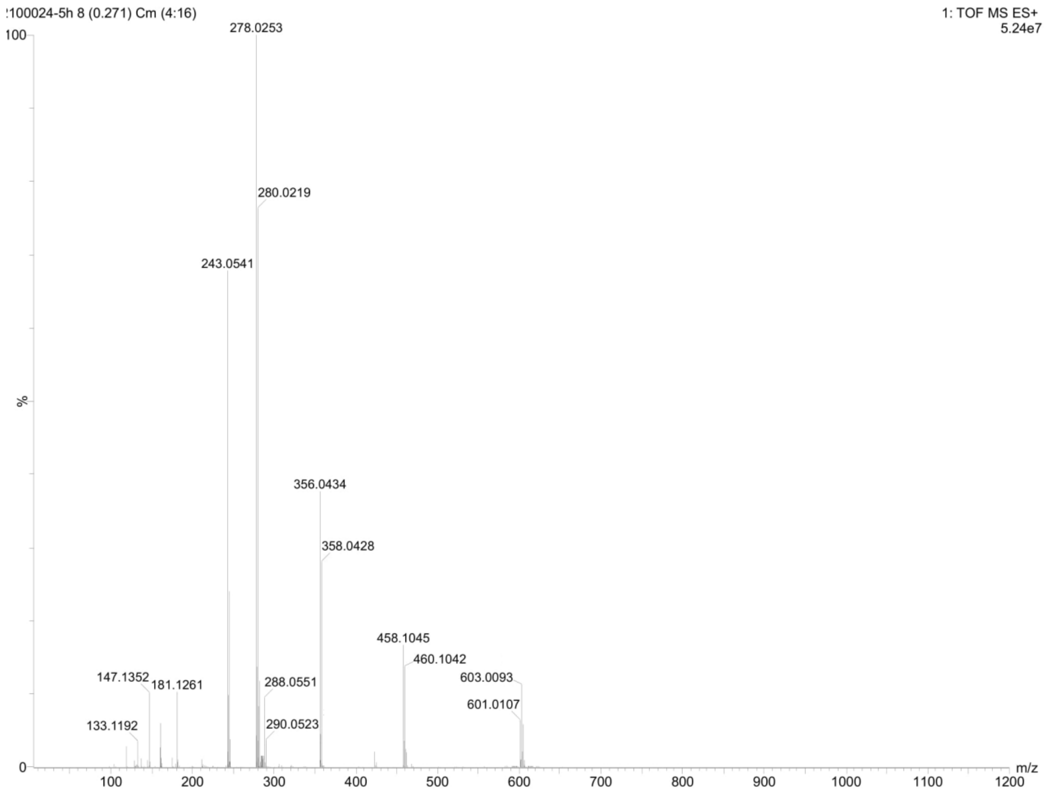

Figure S17: Mass spectrum of complex **C2.**

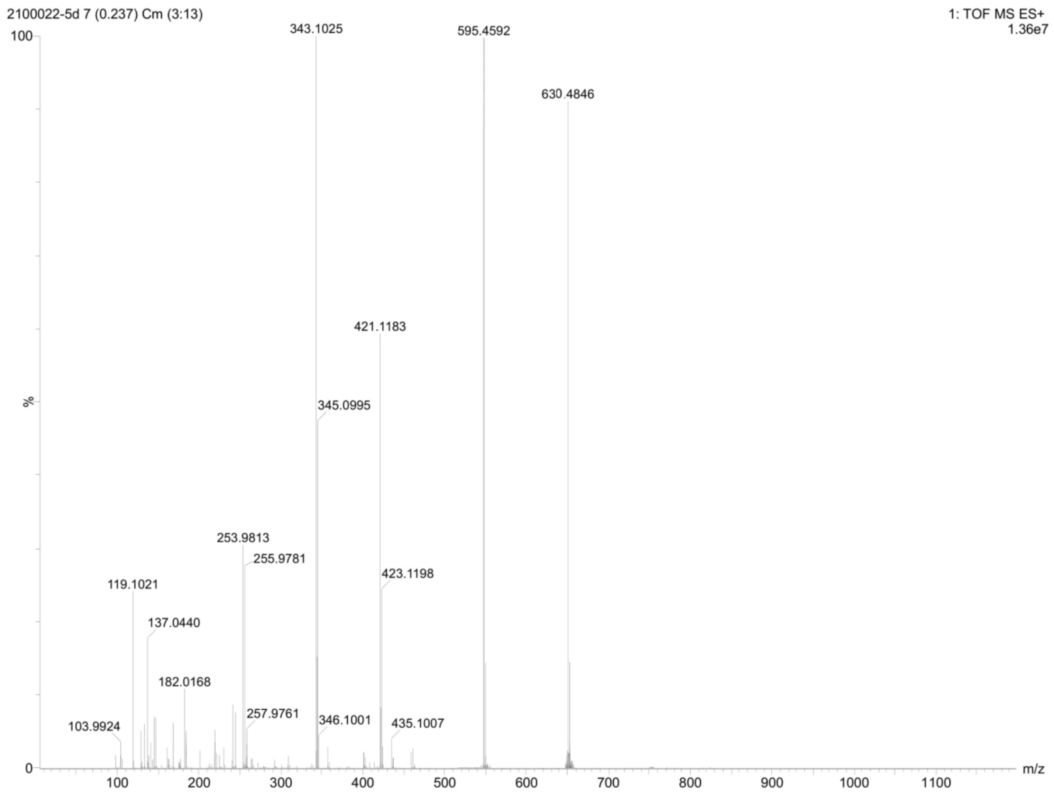

Figure S18: Mass spectrum of complex **C3.**

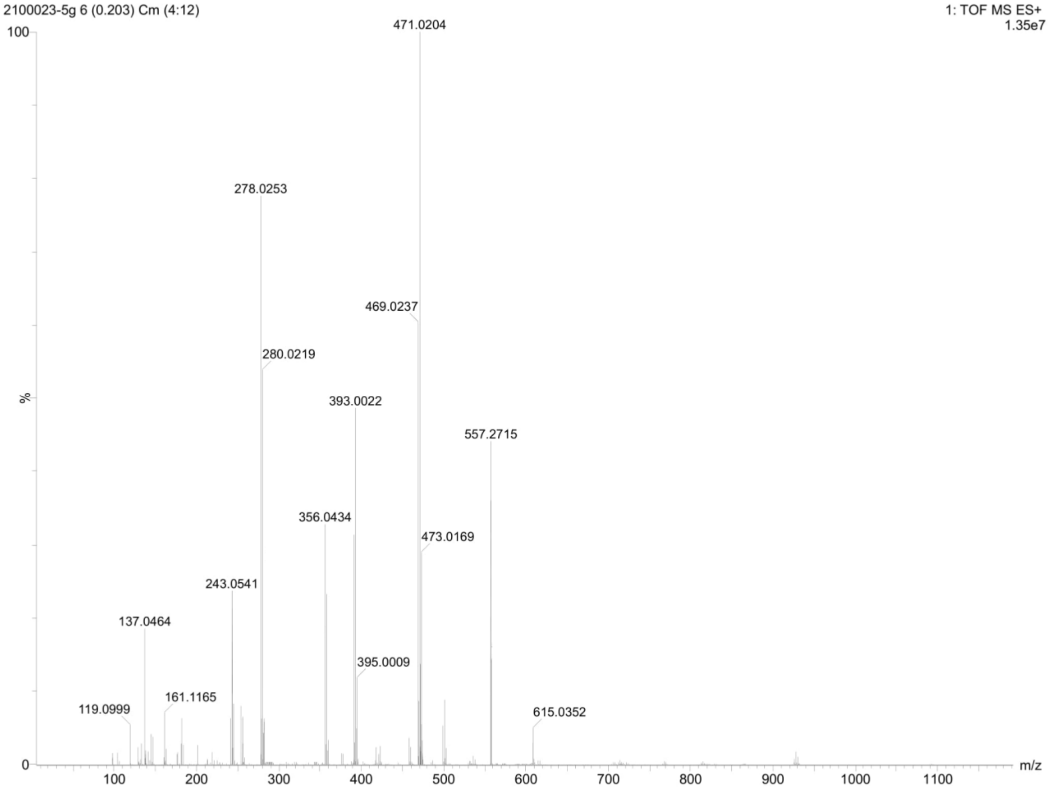

Figure S19: Mass spectrum of complex **C4.**

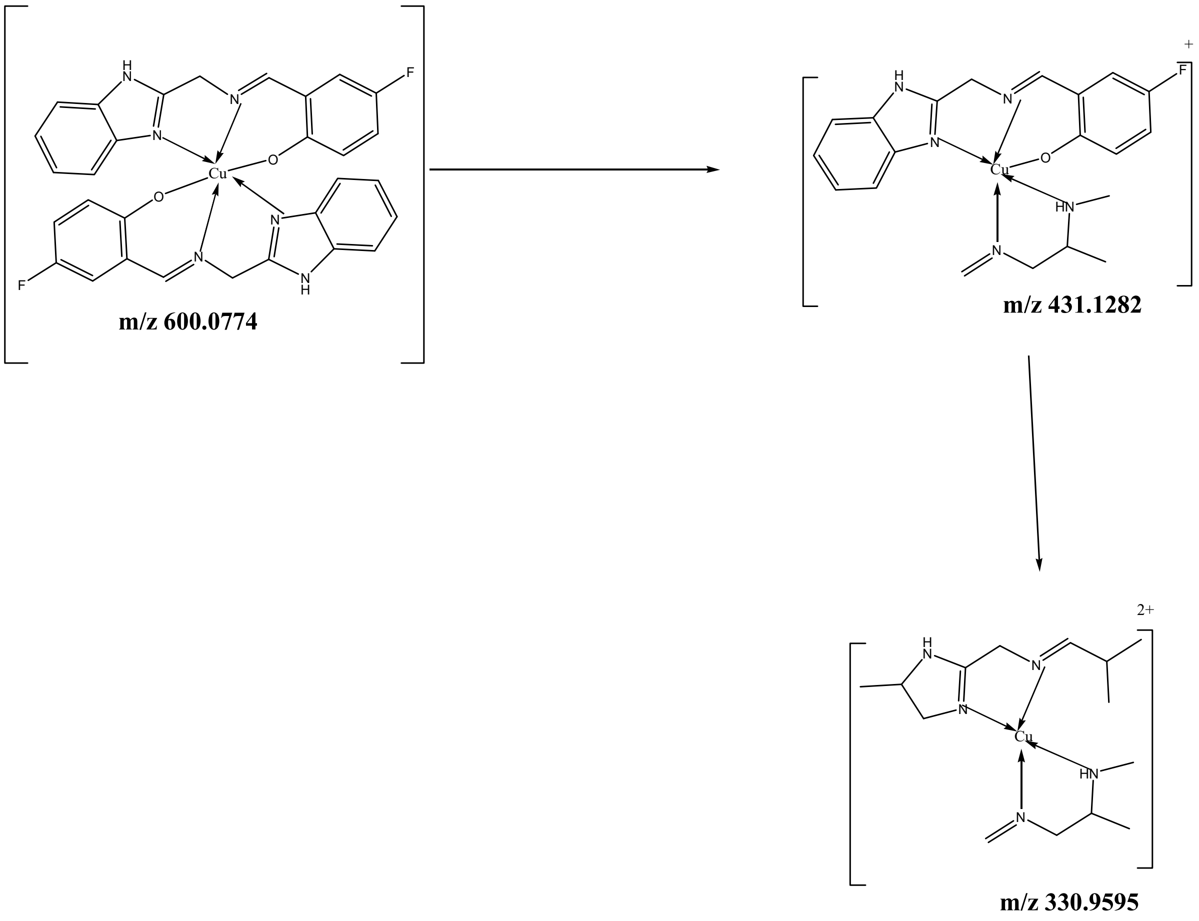

Figure S20: Fragmentation of complex **C1.**

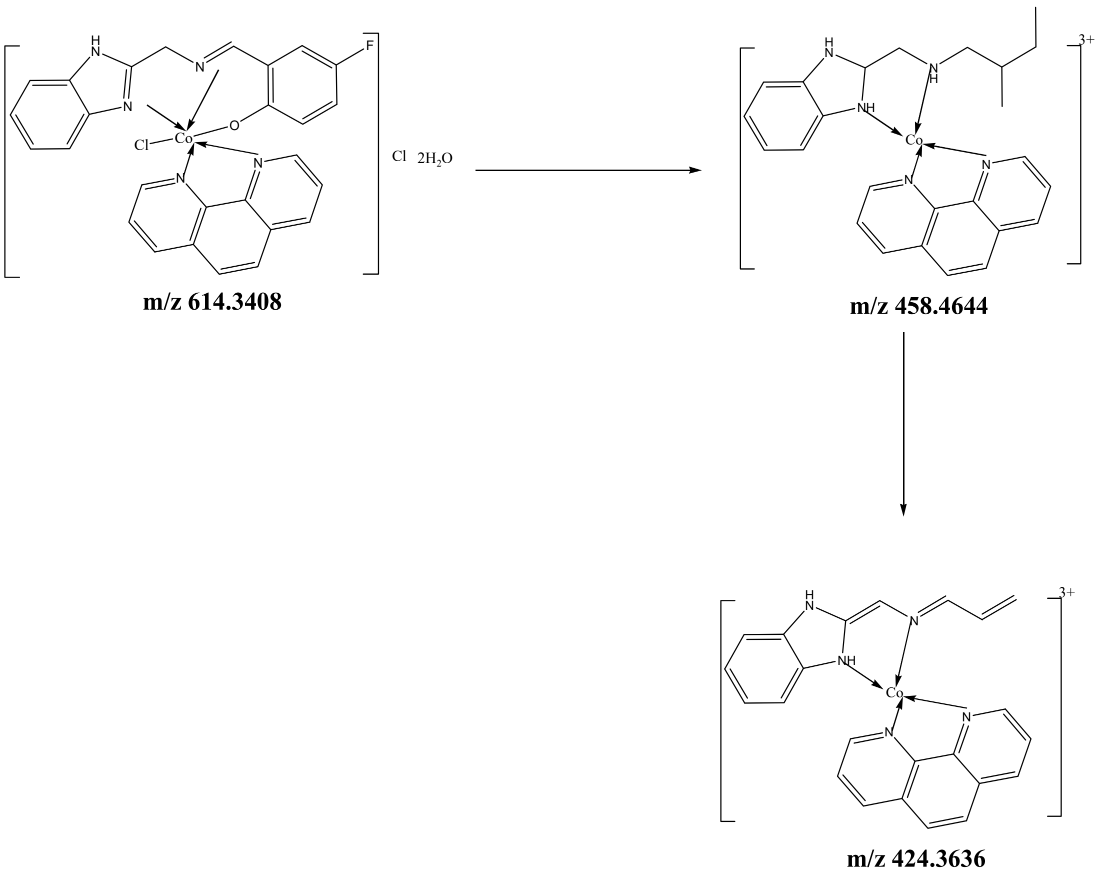

Figure S21: Fragmentation of complex **C4.**

Table S1: Molar conductance of **C1**, **C2**, **C3** and **C4**.

**Complexes**	C1	C2	C3	C4
**Conductance** in μS	00.00	00.00	95.0	64.0

Table S2: IC50 values of HBMF and its complexes.

**IC_{50} Value of HBMF and its complexes (C1-C4) against various cell lines**				
**Compound**	**A549**	**EAC**	**SIHA**	**NIH3T3**
**HBMF**	54.21±0.4	48±0.3	53.4±0.7	52.6±0.4
C1	24.2±0.9	23.4±0.2	25.6±0.3	24.1±1.0
C2	28.1±1.3	25.1±1.2	27.9±0.9	27.2±0.8
C3	14.8±0.8	13.4±0.2	14.2±0.8	15.9±0.4
C4	7.8±0.6	6.9±0.5	7.8±0.2	7.5±0.4
5-FU	7.2±0.5	6.1±1.8	6.2±0.7	7.8V0.4

&

Table S3: IC50 values of Haematological and serum profiles.

**Hematological and Serum profile parameters**	**Normal mice**	**Treated mice with C4**
Alkaline Phosphatase (IU/L)	136.78±1.30	134.45±2.1
Creatinine (mg/dl)	0.39±1.3	0.43±1.25
Urea (mg/dl)	48±2.2	43±2.3
RBC (10^6/μl)	5.7±0.5	5.4±0.6
WBC (10^6/μl)	3.56±2.3	3.5±2.4

Table S4: IC50 values of various parameter as displayed in the below Table.

**Parameters**	Control	**6d** (50 mg/kg. b.w)	**6d**(75 mg/kg.b.w)
Repressed tumor volume in gram	14.43±1.3	05.96±0.6	04.97±0.4
Decrease in ascites secretion in ml	13.03±1.1	07.94±0.7	06.99±0.5
Reduction in tumor cell proliferations [Number of cells × 10^6/(ml)]	80.07±1.8	66.00±1.4	58.07±1.1
Kaplan-Meier showing the extended survivability in number of days	12.00±1.1	19.96±0.7	27.93±0.3
